# Apoptotic priming is defined by the dynamic exchange of Bcl-2 proteins between mitochondria and cytosol

**DOI:** 10.1038/s41418-022-01013-z

**Published:** 2022-05-18

**Authors:** Louise E. King, Ricardo Rodriguez-Enriquez, Robert Pedley, Charlotte E. L. Mellor, Pengbo Wang, Egor Zindy, Michael R. H. White, Keith Brennan, Andrew P. Gilmore

**Affiliations:** 1 Wellcome Trust Centre for Cell-Matrix Research, University of Manchester, Manchester, UK; 2 Division of Cancer Sciences, University of Manchester, Manchester, UK; 3 Division of Molecular and Cellular Function, Faculty of Biology, Medicine and Health, Manchester Academic Health Sciences Centre, University of Manchester, Manchester, UK; 4 Present Address: Institute for Genetics, CECAD Research Center, University of Cologne, Cologne, Germany; 5 Present Address: Cell & Molecular Biology, St. Jude Children’s Research Hospital, Memphis, TN USA; 6 Present Address: Cancer Research UK Manchester Institute, Manchester, UK; 7 Present Address: Center for Microscopy and Molecular Imaging (CMMI), Université libre de Bruxelles (ULB), Gosselies, B-6041 Belgium

**Keywords:** Cell biology, Cancer

## Abstract

Apoptosis is regulated by interactions between the BH3-only and multi-domain Bcl-2 family proteins. These interactions are integrated on the outer mitochondrial membrane (OMM) where they set the threshold for apoptosis, known as mitochondrial priming. However, how mitochondrial priming is controlled at the level of single cells remains unclear. Retrotranslocation of Bcl-XL has been proposed as one mechanism, removing pro-apoptotic Bcl-2 proteins from the OMM, thus reducing priming. Contrary to this view, we now show that Bcl-XL retrotranslocation is inhibited by binding to its BH3-only partners, resulting in accumulation of these protein complexes on mitochondria. We find that Bcl-XL retrotranslocation dynamics are tightly coupled to mitochondrial priming. Quantifying these dynamics indicates the heterogeneity in priming between cells within a population and predicts how they subsequently respond to a pro-apoptotic signal.

## Introduction

Bcl-2 proteins regulate apoptosis through mitochondrial outer membrane permeabilisation (MOMP). The relative proximity of a cell to MOMP, termed mitochondrial priming, determines whether a cell will undergo apoptosis in response to an insult, can predict how tumours respond to chemotherapy [1, 2], and represents a key cancer hallmark [3]. A complex interplay of interactions between different Bcl-2 proteins dictates whether the threshold for MOMP is passed. The Bcl-2 family comprises: pro-apoptotic Bax and Bak that permeabilise mitochondria; anti-apoptotic proteins like Bcl-XL and Bcl-2, which suppress MOMP; and BH3-only proteins that regulate the activities of the others in response to damage signals [4, 5]. Some BH3-only proteins (Bid, Bim, Puma) directly activate Bax and Bak, whereas others, termed sensitisers (e.g., Bad and Noxa), only bind anti-apoptotic Bcl-2 proteins. Anti-apoptotic Bcl-2 proteins bind and sequester activator BH3-only proteins, as well as directly inhibiting Bax and Bak. Sensitisers are proposed to displace activator BH3-only proteins, or Bax and Bak, to initiate MOMP. These interactions between Bcl-2 proteins occur through conserved BH3-domains, which bind within a groove on the surface of their anti-apoptotic partners. These interactions, largely defined in vitro [6], form the rationale behind BH3-mimetics like venetoclax [7]. However, how Bcl-2 protein interactions are coordinated in time and space within a live cell remains unclear.

Mitochondrial priming can be measured experimentally by BH3-profiling [8, 9]. When applied to primary cancer cells, BH3-profiling can predict whether patients will respond to chemotherapy [1, 10]. Similarly, BH3-mimetics selectively kill primed cancer cells *via* binding to specific anti-apoptotic Bcl-2 proteins [11–13]. However, cancer cells show considerable intra-line heterogeneity in their sensitivity to apoptosis [1, 14–17]. Determining how full-length Bcl-2 proteins interact with one another in live cells is important for understanding this heterogeneity. This is particularly relevant as several studies found that the interactions between full-length Bcl-2 proteins and BH3-mimetics in intact cells can differ from those measured in vitro [18–21].

Here we measure the subcellular dynamics of Bcl-2 proteins to interrogate single cell heterogeneity in mitochondrial priming. Bcl-2 family proteins constantly shuttle between mitochondria and the cytosol [22, 23]. For example, Bax retrotranslocation slows in response to reduced kinase signalling, increasing its accumulation on mitochondria to increase priming [23]. Restoring signalling increased Bax retrotranslocation, allowing cells to rapidly adjust priming in response to a dynamic signalling landscape. Bcl-XL and Bcl-W also retrotranslocate [24–26], which has been proposed to remove pro-apoptotic Bcl-2 proteins from mitochondria [22]. However, membrane localisation of Bcl-XL strengthens its binding to BH3-proteins in live cells, correlating with increased priming [20]. Here we have examined the subcellular dynamics of anti-apoptotic Bcl-2 proteins in live cells using fluorescence recovery after photobleaching (FRAP) and fluorescence loss after photoactivation (FLAP). Although Bcl-XL constitutively targets to mitochondria, it is rapidly retrotranslocated back to the cytosol in unprimed cells. BH3-only protein binding inhibits Bcl-XL retrotranslocation, stabilising a mitochondrial complex in primed cells. Interestingly, GFP-Bcl-XL retrotranslocation showed considerable variation between single cells in a population. This variation in Bcl-XL subcellular dynamics predicted the response of cell population subsequently exposed to an apoptotic signal.

## Results

### Mitochondrial targeting is required for anti-apoptotic proteins to suppress BH3-dependent apoptosis

Bax and Bak constitutively retrotranslocate from mitochondria, although with markedly different kinetics [22, 23, 27]. Anti-apoptotic Bcl-2 proteins also shuttle between the mitochondria and the cytosol, removing pro-apoptotic binding partners from the OMM [22]. However, contradictory data suggest that the interaction between Bax and Bcl-XL actually co-stabilises them on mitochondria [23]. To clarify this discrepancy, we examined the role of retrotranslocation in anti-apoptotic Bcl-2 protein function.

**Fig. 1:**
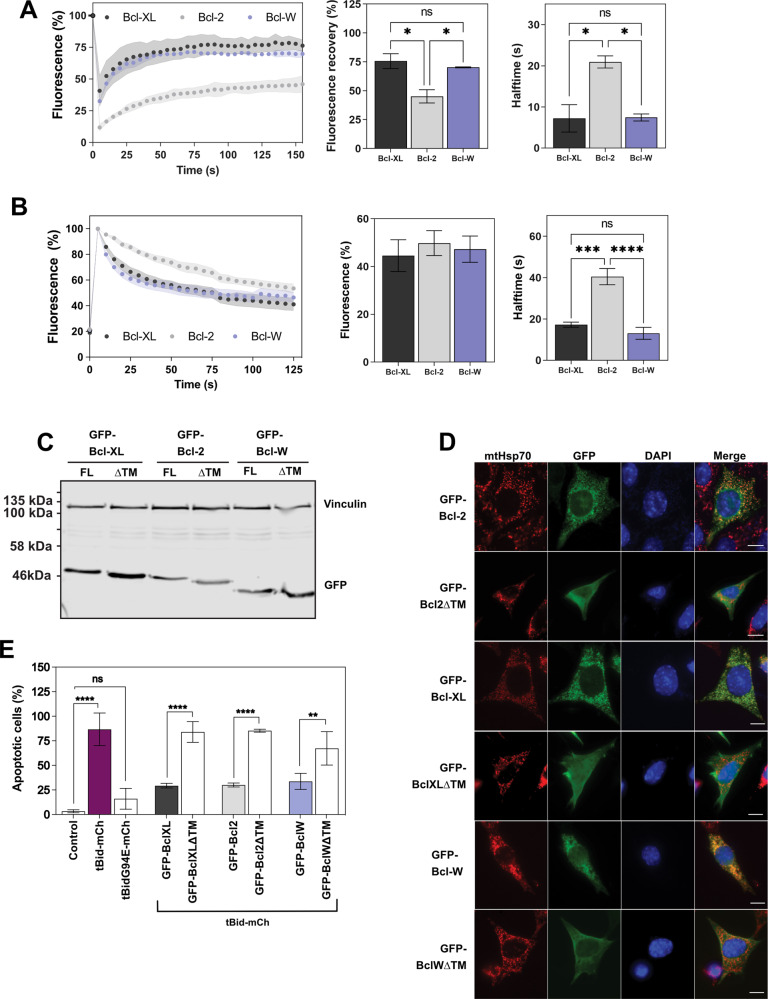
Mitochondrial stabilisation of anti-apoptotic Bcl-2 proteins is required for their function. **A** DKO MEFs transiently expressing GFP-tagged Bcl-XL, Bcl-2 or Bcl-W were subjected to FRAP analysis, and the fluorescence recovery within the photobleached ROI measured. The percentage and halftime of recovery were calculated. Data are mean of two independent experiments, *n* = 25–30 cells per condition. Error bars represent standard deviation and data was analysed by one-way ANOVA and Tukey’s post hoc test. * = *p* < 0.05. **B** DKO MEFs transiently expressing paGFP-tagged Bcl-XL, Bcl-2 or Bcl-W were subjected to FLAP analysis. Fluorescent intensity was analysed within the ROI and initially normalised to 100% post-photoactivation. Data are mean of three independent experiments, *n* = 40 cells per condition. Error bars represent standard deviation and data was analysed by one-way ANOVA and Tukey’s post hoc test. *** = *p* < 0.0005, **** = *p* < 0.00005. **C** Wild type MEFs transiently expressing the indicated FL or truncated (ΔTM) Bcl-2 proteins were lysed and expression levels analysed via Western blot. Expression levels were compared by immunoblotting, using anti-GFP or anti-vinculin loading control. **D** Localisation of the indicated FL and ΔTM Bcl-2 proteins were analysed via immunofluorescence. Cells were immunostained with anti-GFP and anti-mitochondrial Hsp70 (mtHsp70). All three ∆TM variants were exclusively cytosolic. Scale bar represents 10 µm. **E** Wildtype MEFs transiently expressing the indicated FL or ∆TM variants of Bcl-XL, Bcl-2, and Bcl-W in combination with tBid-mCh were assayed for anti-apoptotic function. Control cells were transfected with only mCh-tBid or mCh-tBidG94E. Cells were stained for anti-mCherry, anti-active caspase 3, and DAPI. Only cells positively transfected with mCh-tBid were analysed. Data are mean of three independent experiments. Error bars represent standard deviation and data was analysed by one-way ANOVA and Šídák’s post hoc test. ** = *p* < 0.005, *** = *p* < 0.0005, **** = *p* < 0.00005.

Bax/Bak double knockout (DKO) MEFs were transiently transfected with full-length fluorescent-protein tagged Bcl-XL, Bcl-W, or Bcl-2. Fluorescent recovery after photobleaching (FRAP) allows rapid analysis of protein mobility within live cells. Mitochondrial GFP-Bcl-XL fluorescence recovered rapidly within the photobleached region of interest (ROI), indicating that the protein was dynamically exchanging between membrane bound and soluble fractions (Fig. 1A and Fig. S1A). Mitochondrial Bcl-W fluorescence recovered with similar kinetics to Bcl-XL. In contrast, Bcl-2 recovered significantly slower with a larger immobile fraction on mitochondria than either Bcl-XL or Bcl-W. We confirmed this using photo-activatable (PA) GFP-tagged Bcl-XL, Bcl-W, and Bcl-2, measuring FLAP (Fig. 1B; Fig. S1B; Supplementary Movies 1–3). Thus, like Bax vs. Bak, Bcl-XL and Bcl-W show distinct retrotranslocation dynamics compared to Bcl-2.

Anti-apoptotic Bcl-2 proteins lacking their C-terminal transmembrane (TM) regions still bind BH3-domain peptides within their exposed hydrophobic pocket [6]. However, there are conflicting reports on whether their anti-apoptotic function requires mitochondrial localisation [20, 28]. We generated variants of GFP-Bcl-2, GFP-Bcl-W, and GFP-Bcl-XL in which the transmembrane helix had been deleted (∆TM) by inserting stop codons as indicated (Fig. S1C). All the ∆TM variants expressed at similar levels to the full-length (FL) proteins (Fig. 1C). However, they were cytosolic whereas the FL variants co-localised with mitochondria (Fig. 1D). To test functionality, we transiently expressed in wild-type MEFs GFP-tagged FL or ∆TM Bcl-2, Bcl-W, or Bcl-XL, along with mCherry-tagged tBid (tBid-mCh), and quantified apoptosis in cells positive for both (Fig. 1E). tBid-mCh alone induced apoptosis in around 80% of cells, whereas the inactive variant tBidG94E-mCh did not. Co-expression of FL Bcl-2, Bcl-W, or Bcl-XL suppressed tBid-mCh induced apoptosis. However, the ∆TM variants were significantly impaired in their ability to suppress tBid-mCh. Thus, the ability of Bcl-2, Bcl-XL, and Bcl-W to associate with mitochondria was necessary for their anti-apoptotic activity in cells, despite their very different retrotranslocation dynamics.

### BH3-protein binding to Bcl-XL and Bcl-W reduces mitochondrial retrotranslocation

**Fig. 2:**
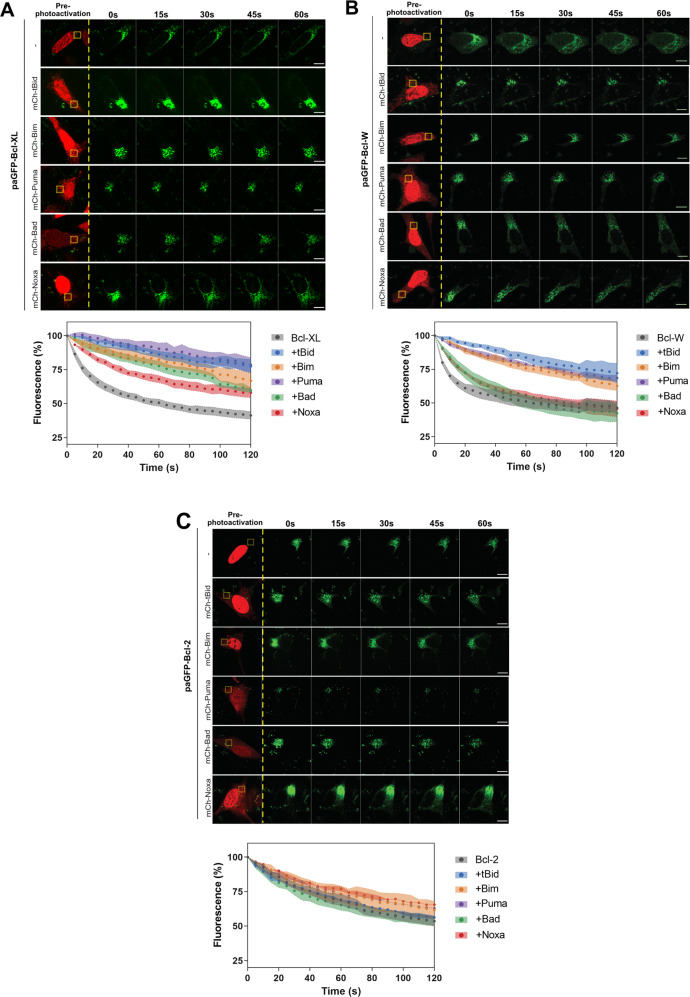
Bcl-XL is stabilised on mitochondria by BH3-domain-specific interactions. **A** Bax/Bak DKO MEFs transiently expressing photoactivatable paGFP-Bcl-XL and mCherry-tagged BH3-proteins were photoactivated in the yellow ROI and imaged every 5 s. Fluorescent intensity was analysed within the ROI and initially normalised to 100% post-photoactivation. The data were fitted to a one-phase exponential dissociation and show the mean of three independent experiments, 40 cells per condition +/− SEM. Cells co-expressed mRFP-H2B. See Supplementary Movies 1–3. **B** Bax/Bak DKO MEFs transiently expressing paGFP-Bcl-W and mCherry-tagged BH3-proteins were photoactivated in the yellow ROI and imaged every 5 s. Images were analysed as in A. Data are the mean of two independent experiments, *n* = 30 cells per condition. Error bars = SEM. **C** Bax/Bak DKO MEFs transiently expressing paGFP-Bcl-2 and mCherry-tagged BH3-proteins were photoactivated in the yellow ROI and imaged every 5 s. Images were analysed as in (**A**). Data are the mean of two independent experiments, *n* = 30 cells per condition. Error bars = SEM. Scale bars represent 10 µm.

Binding between BH3-only and multi-domain anti-apoptotic proteins have largely been defined in vitro [6, 29, 30]. As mitochondrial targeting was required for Bcl-XL, Bcl-W and Bcl-2 to suppress tBid-dependent apoptosis, interactions between them would occur on the OMM. We therefore hypothesised that BH3 binding would decrease retrotranslocation. To test this, we used paGFP-Bcl-XL, paGFP-Bcl-2 and paGFP-Bcl-W to measure retrotranslocation in the presence of mCh-tagged BH3-only proteins. We expressed paGFP-Bcl-XL in DKO MEFs along with H2B-mRFP to identify transfected cells (Fig. 2A; Supplementary Movies 4–8). paGFP-Bcl-XL was co-expressed with mCh-tagged Bad, tBid, Puma, Noxa, or BimEL. Cells expressing both H2B-mRFP and the mCh-BH3 protein (identified by its mitochondrial localisation) were photoactivated within a defined ROI, and the subsequent loss of paGFP-Bcl-XL from that ROI quantified. paGFP-Bcl-XL loss from the photoactivated ROI was markedly reduced in cells co-expressing mCh-tagged tBid, BimEL, Puma and Bad. BH3-peptides corresponding to these BH3-proteins all bind Bcl-XL in vitro [6]. mCh-Noxa, whose BH3-peptide does not bind Bcl-XL in vitro, had less effect on paGFP-Bcl-XL retrotranslocation than the other proteins, but still increased paGFP-Bcl-XL mitochondrial stability compared to cells expressing paGFP-Bcl-XL alone.

We asked if reduced Bcl-XL retrotranslocation was dependent on canonical BH3-domain binding. We co-expressed in DKO MEFs GFP-Bcl-XL and mCh-BH3 proteins with substitutions within the BH3-domain that reduce binding within the hydrophobic pocket. Both tBid-mCh and mCh-BimEL reduced GFP-Bcl-XL FRAP (Fig. S2A, B). However, neither mCh-tBidG94E nor mCh-tBid2A reduced GFP-Bcl-XL FRAP (Fig. S2A). When GFP-Bcl-XL was co-expressed with mCh-BimEL2A, FRAP recovery was slower than Bcl-XL alone, but less so than with WT BimEL (Fig. S2B). This difference in Bcl-XL translocation dynamics between WT and 2A mutants was functional, as WT MEFs transfected with tBid-mCh or mCh-BimEL in the absence of GFP-Bcl-XL underwent significant apoptosis (Fig. S2C). tBid2A did not induce apoptosis, whereas BimEL2A induced apoptosis but not to the level as with WT BimEL, agreeing with data showing that BimEL2A still interacts with Bcl-XL in live cells through a second site [21, 24].

paGFP-Bcl-W showed behaviour distinct to paGFP-BclXL when co-expressed with BH3-only proteins (Fig. 2B). mCh-BimEL, tBid, and Puma all decreased paGFP-Bcl-W retrotranslocation. In vitro studies indicate that Noxa BH3-peptides do not bind Bcl-W, whereas Bad BH3-peptides do [6]. However, the FLAP data here suggested that neither Bad nor Noxa stabilised mitochondrial paGFP-Bcl-W in live cells. paGFP-Bcl-2, being more stable on mitochondria than the other proteins, did not change following co-expression with mCh-tagged BH3-only proteins (Fig. 2C).

These data indicate that BH3-only protein binding to anti-apoptotic proteins slows retrotranslocation rates from mitochondria, and the binding specificity of BH3-only proteins for full-length anti-apoptotic Bcl-2 proteins in live cells gives a different picture from that determined using BH3-peptides and truncated proteins in vitro.

### Reduced Bcl-XL retrotranslocation correlates with removal of growth factor signalling

**Fig. 3:**
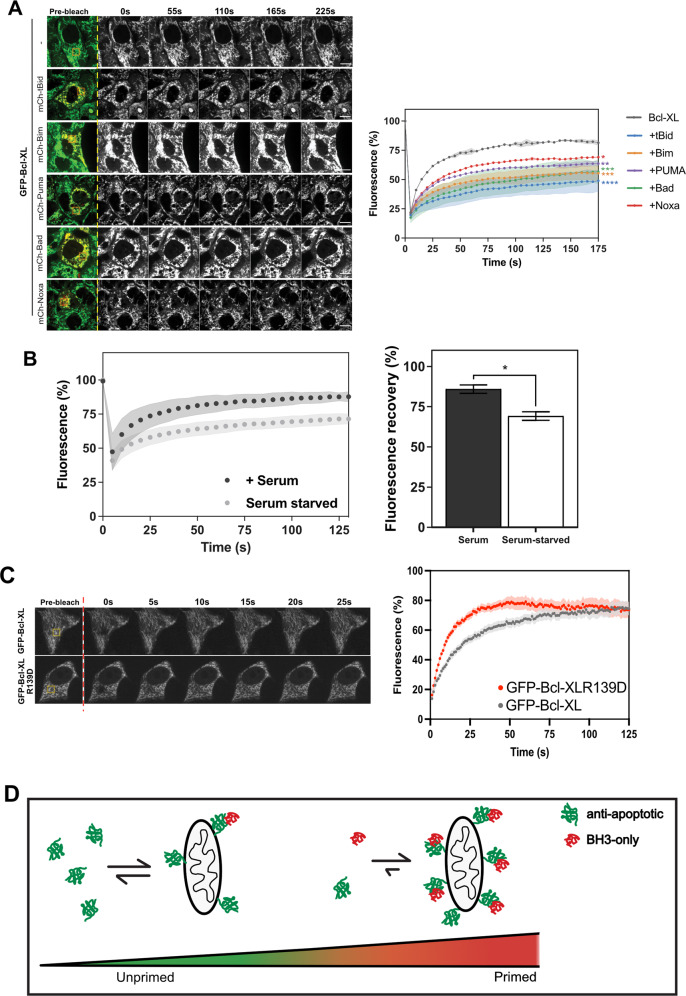
Serum starvation reproduces BH3-protein dependent changes in GFP-Bcl-XL retrotranslocation. **A** MCF10A mammary epithelial cells stably expressing GFP-Bcl-XL and transiently transfected with the indicated mCh-BH3 proteins, were subjected to FRAP analysis. GFP-Bcl-XL was photobleached in the yellow ROI and fluorescent intensity normalised to 100% pre-bleaching. The data were fitted to a one-phase exponential association. Data show the mean of two independent experiments. *n* = 20 cell per condition, +/− SD. Scale bar represents 10 µm. Fluorescence recovery was analysed by one-way ANOVA. * = *p* < 0.05, ** = *p* < 0.01, *** = *p* < 0.005, **** = *p* < 0.001. **B** MCF10As stably expressing GFP-Bcl-XL were cultured in either complete growth medium, or serum-free medium for 7 days before carrying out FRAP analysis. Non-linear regression of the average fluorescence recovery was carried out from the data. Data represents values from three independent experiments, *n* = 75 cells per condition per repeat. Error bars represent standard deviation and data was analysed via Student’s t-test. * = *p* < 0.05. **C** MCF10A cells expressing either GFP-Bcl-XL or GFP-Bcl-XLR139D were subjected to FRAP analysis as in (**A**). Images were taken every second due to the rapid recovery for the GFP-Bcl-XLR139D variant compared to WT. Fluorescent intensity was normalised to 100% pre-bleaching. The data were fitted to a one-phase exponential association. Data show the mean +/− SEM. **D** Schematic hypothesis showing the proposed accumulation of Bcl-XL on mitochondria in BH3-only protein-bound complexes.

As GFP-Bcl-XL retrotranslocation was inhibited by the broadest range of BH3-only proteins, we hypothesised that we could use it to assess their activity in live cells. We generated MCF10A mammary epithelial cells stably expressing GFP-Bcl-XL using lentiviral transduction. These were then transiently transfected with mCh-tagged Bad, tBid, Puma, Noxa, or BimEL, and FRAP analysis performed (Fig. 3A). The results supported those obtained with paGFP-Bcl-XL in DKO MEFs, with fluorescence recovery of GFP-Bcl-XL significantly reduced in the presence of tBid, BimEL, Puma, and Bad, with mCh-Noxa the least effective.

The transient expression of BH3-only proteins would mimic the mitochondrial priming observed when cells are exposed to a stress that would increase their activity. We therefore hypothesised that there would be reduced GFP-Bcl-XL retrotranslocation in cells subjected to conditions such as reduced growth factor receptor (GFR) signalling, which would increase priming but not by itself induce apoptosis. MCF10A cells stably expressing GFP-Bcl-XL were cultured in either complete growth medium or in serum and growth factor (GF)-free conditions before FRAP analysis (Fig. S3A). Starved cells showed a significant decrease in Bcl-XL retrotranslocation, similar to that observed following transient expression of BH3 proteins (Fig. 3B, cf. Fig. 3A). Based on this result, we conjectured that endogenous BH3-only proteins set the basal level of GFP-Bcl-XL retrotranslocation, and this should then increase in the absence of any BH3 binding. To test this, we used GFP-Bcl-XLR139D, which has impaired BH3-domain binding [31]. As expected, GFP-Bcl-XLR139D exhibited faster retrotranslocation than WT in MCF10A cells in the absence of any apoptotic signal (Fig. 3C).

Together, these data indicate that Bcl-XL is rapidly exchanged between mitochondria and the cytosol in non-apoptotic cells (Fig. 3D). Membrane-associated Bcl-XL is stabilised through BH3-dependent interactions, with increased stabilisation at the OMM equating with increased BH3-only protein activity in cells.

### Bcl-XL retrotranslocation indicates dynamic changes in BH3-only protein activity within live cells

**Fig. 4:**
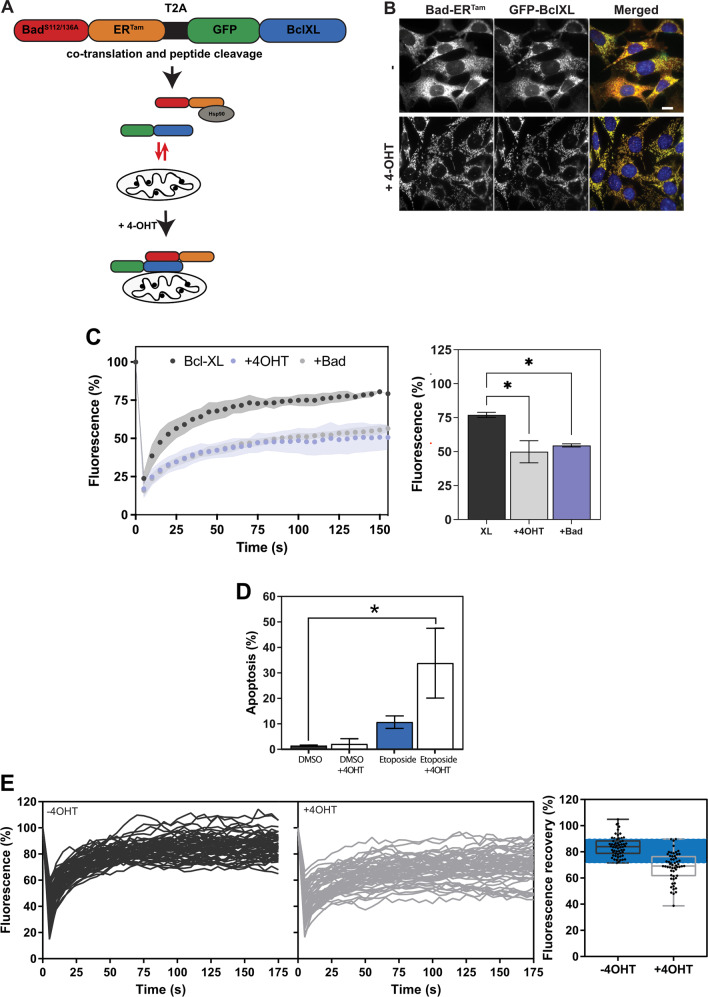
Bcl-XL retrotranslocation indicates dynamic changes in BH3-only protein activity within live cells. **A** Schematic showing inducible BadER^Tam^ coexpression with GFP-Bcl-XL. The construct is expressed from a lentiviral vector incorporating a self-cleaving T2A peptide, allowing co-expression of both proteins from the same transcript. In the absence of 4-hydroxy tamoxifen (4-OHT), BadER^Tam^ is inactive in the cytosol, bound to Hsp90. In the presence of 4-OHT, BadER^Tam^ is released, allowing binding to Bcl-XL at the OMM. **B** Cells stably expressing BadER^Tam^/GFP-BclXL were treated with ethanol alone (-) or 4-OHT and immunofluorescence carried out. Cells were stained with antibodies against ER and GFP. Scale bar represents 10 µm. **C** MCF10A cells stably expressing BadER^Tam^/GFP-BclXL were treated with either ethanol or 4-OHT and FRAP analysis carried out as in Fig. 3. For comparison, stable MCF10A BadER^Tam^/GFP-BclXL cells were transiently transfected with a plasmid expressing mCh-Bad. The addition of 4-OHT significantly reduced GFP-Bcl-XL dynamics, and this was indistinguishable from that seen in cells expressing mCh-Bad. Data represent the mean of two independent experiments, *n* = 30 cells per condition. Error bars represent standard deviation and data was analysed by one-way ANOVA with Tukey’s post hoc test. * = *p* < 0.05. **D** Stable BadER^Tam^/GFP-BclXL MCF10A cells treated with combinations of 4-OHT and/or Etoposide for 24 h and apoptosis quantified via immunofluorescence for active caspase 3 and DAPI. Values represent data from three independent experiments. Error bars represent SEM and data was analysed by one-way ANOVA with Tukey’s post hoc test. * = *p* < 0.05. **E** MCF10A cells expressing BadER^Tam^/GFP-BclXL were treated with either ethanol (-4OHT) or 4-OHT and FRAP performed as above on 60 cells under each condition. FRAP curves for each individual cell are shown, with the distribution of percentage recovery.

As decreased Bcl-XL FRAP dynamics correlated with reduced GFR signalling or expression of BH3-only proteins, we conjectured that this would allow monitoring of dynamic changes in BH3-only protein activity in live cells. To test this, we modelled BH3-only activity using a conditionally active version of Bad, BadER^Tam^. Bad links GFR signalling to priming in MECs [32, 33] but, being a sensitiser, it cannot activate MOMP. Phosphorylation at serine 112 and 136 sequesters Bad in the cytosol *via* 14.3.3 binding [34]. MCF10A cells grown in serum/GF-free conditions showed reduced phosphorylation and increased mitochondrial association of endogenous Bad (Fig. S3B, C), correlating with slower Bcl-XL retrotranslocation (Fig. 3B). To mimic this mechanism, we fused Bad with the oestrogen receptor (ER) hormone-binding domain and substituted serines 112 and 136 for alanine, generating BadER^Tam^. We stably infected MCF10A cells (which are ER negative) with a lentivirus co-expressing BadER^Tam^ and GFP-Bcl-XL (Fig. 4A, B). BadER^Tam^ was cytosolic, but became mitochondrial following the addition of 4-hydroxytamoxifen (4-OHT), which was accompanied by increased mitochondrial association of GFP-Bcl-XL (Fig. 4B). Thus, this allowed us to manipulate Bad-dependent mitochondrial priming using an artificial stimulus independent of other BH3-only proteins.

We performed FRAP analysis on MCF10A BadER^Tam^/GFP-Bcl-XL cells in the presence or absence of 4-OHT, or expression of mCh-Bad. 4-OHT reduced GFP-Bcl-XL retrotranslocation similar to mCh-Bad (Figs. 4C and S4A) or serum/GF starvation (cf. Fig. 3B). 4-OHT did not reduce GFP-Bcl-XL FRAP in the absence of BadER^Tam^ (Fig. S4B). BadER^Tam^ effects on retrotransloction were reversed following 4-OHT washout (Fig. S4C). These changes in Bcl-XL dynamics were linked to mitochondrial priming, as MCF10A BadER^Tam^/GFP-Bcl-XL cells pre-treated with 4-OHT were more susceptible to subsequent etoposide treatment than control cells (Fig. 4D).

Anti-apoptotic Bcl-2 proteins have been suggested to employ two distinct modes of MOMP inhibition, based on whether they are sequestering activator BH3-only proteins (MODE1) or pore forming effectors Bax and Bak (MODE2) [35]. We asked if Bcl-XL FRAP dynamics were associated with either MODE1 or MODE2 inhibition. Bax/Bak DKO MEFs stably expressing BadER^Tam^/GFP-Bcl-XL were transiently transfected with either tBid-mCh (to mimic MODE1 inhibition), or mCh-Bak which preferentially binds Bcl-XL (MODE2) [36]. tBid-mCh, but not tBid2A-mCh, stabilised mitochondrial GFP-Bcl-XL (Fig. S5A). Simultaneous mCherry FRAP indicated that tBid-mCh was sequestered by the GFP-Bcl-XL, consistent with MODE1 inhibition (Fig. S5B). 4-OHT activation of BadER^Tam^ displaced tBid-mCh, increasing mCherry FRAP. In contrast, MODE2 inhibition displayed a distinct profile. mCh-Bak did not stabilise mitochondrial GFP-Bcl-XL to the extent seen with BH3-only proteins (Fig. S5C). Activated BadER^Tam^ increased stabilisation of mitochondrial GFP-Bcl-XL in mCh-Bak expressing cells, suggesting that Bad might preferentially compete for Bcl-XL binding in MODE2. mCh-Bak itself retrotranslocates slowly [23, 27], and was not changed by BadER^Tam^ (Fig. S5D).

These data indicate that the dynamics of Bcl-XL retrotranslocation are directly linked to apoptotic priming by BH3-only proteins.

### Heterogeneity in Bcl-XL dynamics is a cell intrinsic property that predicts single-cell response to pro-apoptotic signals

We were intrigued to observe significant cell-to-cell variation in GFP-Bcl-XL dynamics, with overlap between untreated and 4-OHT-treated conditions (Fig. 4E). Quantification of endogenous Bad S112 phosphorylation showed similar cell-to-cell variation, suggesting inherent heterogeneity in BH3-only protein-dependent priming in a population (Fig. S6A). Thus, a proportion of cells in each condition would fit either a primed or unprimed profile and this might be quantified through GFP-Bcl-XL FRAP dynamics.

**Fig. 5:**
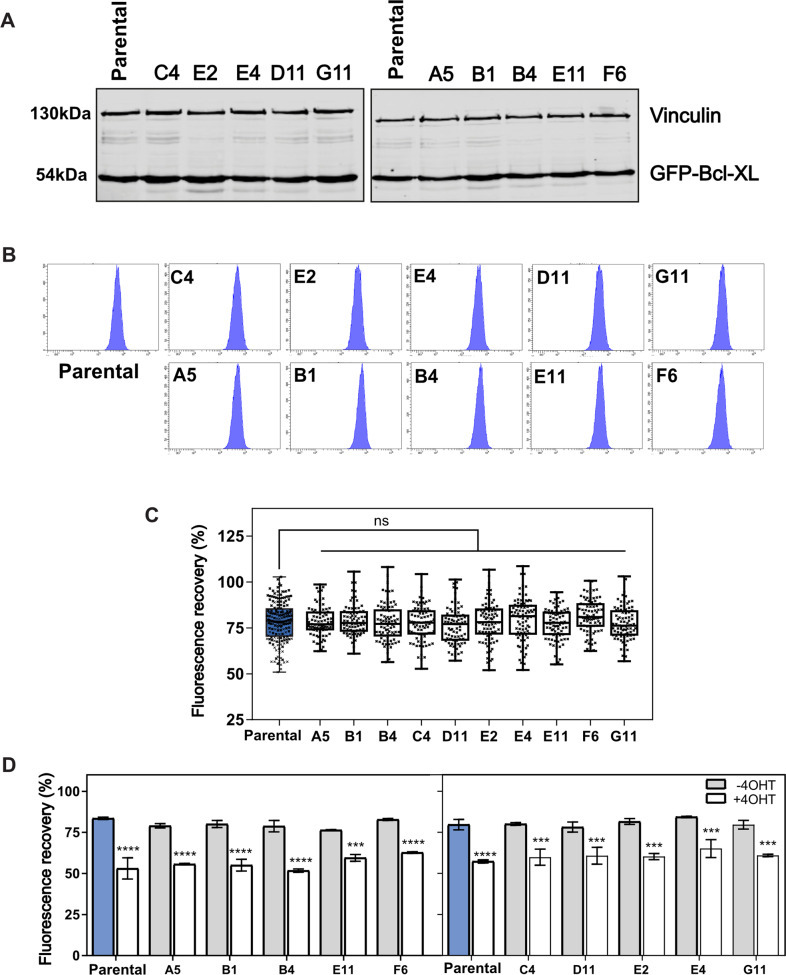
Single cell variations in GFP-Bcl-XL dynamics are cell autonomous. **A** Single cell clones of MCF10As stably expressing BadER^Tam^ and GFP-Bcl-XL were generated. Ten independent clones were subject to Western blot analysis with anti-Bcl-XL and anti-vinculin, and expression levels were compared with the polyclonal parental cells. **B** Flow cytometry analysis of GFP-Bcl-XL expression in the polyclonal parental cells and single cell clones from (**A**). **C** FRAP analysis performed on the parental and clonal cell populations in (**A**), showing single cell data on fluorescence recovery. There were no significant differences in the single cell heterogeneity between the single cell clones. Data show the mean of ~60 cells per line. Error bars represent standard deviation and data was analysed by one-way ANOVA with Šídák’s post hoc test. **D** Mean fluorescence recovery for parental and clonal cell populations was calculated for both untreated and 4-OHT treated cells. All populations showed significantly more stable GFP-Bcl-XL after 4-OHT treatment. Values represent data from three independent experiments and error bars represent SD. Data was analysed via one-way ANOVA with Šídák’s post hoc test *** = *p* < 0.005; **** = *p* < 0.001.

The MCF10A GFP-Bcl-XL lines were established by lentiviral infection followed by selecting GFP positive cells by FACS. Although we selected cells within a narrow GFP expression window, it was possible that the heterogeneity in dynamics was due clonal variation. We established ten independent single cell clones from the MCF10A BadER^Tam^/GFP-Bcl-XL cells. Immunoblotting (Fig. 5A) and flow cytometry (Fig. 5B) indicated that all ten clones had similar levels of GFP-Bcl-XL expression, which was also similar to the parental population. GFP-Bcl-XL showed similar mitochondrial localisation in all clones (Fig. S6B). To determine if the variation in dynamics was clonal, we performed FRAP analysis on all ten lines and compared these to the parental line. There were no differences between the GFP-Bcl-XL FRAP kinetics of any of the ten clonal lines and the parental population, with similar variation within each (Figs. 5C and S6C). All clones showed a similar increase in GFP-Bcl-XL mitochondrial stability following BadER^Tam^ activation (Fig. 5D). These data indicate that random viral integration into the genome and clonal variation is not a cause of the variation in GFP-Bcl-XL dynamics seen. The heterogeneity in Bcl-XL retrotranslocation within the population is therefore a cell intrinsic property.

**Fig. 6:**
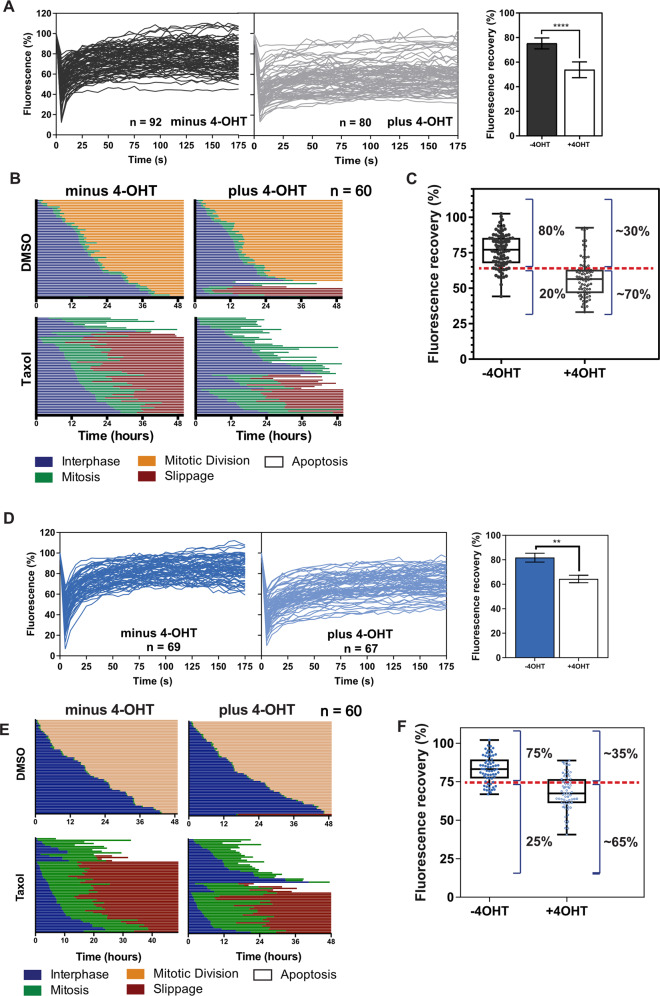
Single cell heterogeneity in GFP-Bcl-XL dynamics predicts a cell population’s response to Taxol. **A** MDA-MB-231 cells stably expressing BadER^Tam^ and GFP-Bcl-XL were treated with ethanol alone (minus 4-OHT) or 4-hydroxy tamoxifen (plus 4-OHT). GFP-Bcl-XL was photobleached and images captured every five seconds. Single cell data are presented from the indicated number of cells per condition. The mean fluorescence recovery of the population calculated from a non-linear regression curve fit from three independent experiments. Error bars represent standard deviation and data was analysed via Student’s t-test. **** = *p* < 0.001. **B** MDA-MB-231 cells stably expressing BadER^Tam^ and GFP-Bcl-XL were treated with the indicated combinations of 4-OHT and Taxol, and images captured every 15 min for 50 h to determine cell fate. Sixty individual cells from two independent experiments were tracked and the times they entered mitosis indicated in green. The subsequent fate of each cell was followed and the timing is indicated (mitotic division, apoptosis, slippage). **C** MDA-MB-231 cells treated as in (**A**), and analysed by FRAP plotted as a box and whisker plot showing the distribution of *Y*_max_ values for single cell FRAP from four independent experiments. The red line shows the ~20% apoptosis threshold set for the minus 4-OHT cells determined following Taxol treatment. On the right of each graph is shown the proportion of cells above and below the threshold for Taxol induced apoptosis. **D** MCF10A cells stably expressing BadER^Tam^ and GFP-Bcl-XL were treated with ethanol alone (minus 4-OHT) or 4-hydroxy tamoxifen (plus 4-OHT). GFP-Bcl-XL was photobleached and images captured every five seconds. Data presented are the individual cell values from the indicated number of cells per condition. The mean fluorescence recovery was calculated as in (**A**). Error bars represent standard deviation and data was analysed via Student’s t-test. ** = *p* < 0.01. **E** GFP-Bcl-XL-BadER^Tam^ MCF10A cells were treated with the indicated combinations of 4-OHT and Taxol, single cell fate determined as in (**B**). Ninety individual cells from three independent experiments were tracked and the time they entered mitosis indicated in green. **F** MCF10A cells treated as in (**D**), and analysed by FRAP plotted as a box and whisker plot showing the individual *Y*_max_ values. The red line shows the apoptosis threshold set for the minus 4-OHT cells determined following Taxol treatment. On the right of each graph is shown the proportion of cells above and below the threshold for Taxol-induced apoptosis.

Cell populations show varied responses to apoptotic signals [37]. For example, cells arrested in mitosis with Taxol show a range of outcomes, including apoptosis or slippage (exiting mitosis without dividing) [15, 38]. The level of pre-mitotic mitochondrial priming influences the outcome of mitotic arrest. We asked if we could predict this by measuring GFP-Bcl-XL dynamics in a cancer cell population prior to Taxol treatment. We examined the triple negative breast cancer line, MDA-MB-231, which shows a range of responses to Taxol mediated mitotic arrest [39], and generated stable cells co-expressing BadER^Tam^ and GFP-Bcl-XL. FRAP analysis indicated a significant mobile fraction of GFP-Bcl-XL that became stabilised following treatment with 4-OHT (Fig. 6A). As before, there was considerable variation in GFP-BclXL FRAP kinetics between cells, with and without 4-OHT.

To determine how GFP-Bcl-XL dynamics relate to the apoptotic response, MDA-MB-231/BadER^Tam^/GFP-Bcl-XL cells were treated with DMSO or Taxol. We individually tracked single cell fates over 50 h, and determined when each entered mitosis, and whether it subsequently underwent mitosis, apoptosis, or slippage (Fig. 6B). Taxol alone prolonged time in mitosis, but the majority of MDA-MB-231/BadER^Tam^/GFP-Bcl-XL cells underwent slippage, only ~20% of the population undergoing apoptosis. Parental MDA-MB-231 cells had a similar response to Taxol, indicating it is unaffected by the stable expression of GFP-Bcl-XL (Fig. S7). To determine if the threshold for Taxol-induced apoptosis could be predicted based upon GFP-Bcl-XL kinetics, we performed FRAP on MDA-MB-231/BadER^Tam^/GFP-Bcl-XL in the absence of 4-OHT and determined *Y*_max_ for the bottom 20% of cells (Fig. 6C). We transposed this *Y*_max_ threshold onto FRAP data from MDA-MB-231/BadER^Tam^/GFP-Bcl-XL cells following the activation of BadER^Tam^ with 4-OHT. If GFP-Bcl-XL dynamics were predictive of apoptotic priming, we would expect ~70% of 4-OHT treated cells to undergo apoptosis in Taxol. 4-OHT alone did not induce significant apoptosis or impair normal mitotic progression (Fig. 6B). However, both 4-OHT and Taxol resulted in ~70% apoptosis, close to that predicted by pre-treatment FRAP (Fig. 6B, C). Parental MDA-MB-231 cells were not sensitised to Taxol-mediated apoptosis by 4-OHT (Fig. S7).

We repeated our analysis on the MCF10A/BadER^Tam^/GFP-Bcl-XL cells to determine if this prediction held for other cell types. FRAP analysis was performed in the absence or presence of 4-OHT (Fig. 6D). In the absence of Taxol, 4-OHT did not influence the proportion of apoptosis or slippage in the MCF10A cells (Fig. 6F). Approximately 25% of cells underwent apoptosis in Taxol. From this and based on the FRAP kinetics, we predicted ~60% of cells pre-treated with 4-OHTwould die in Taxol (Fig. 6D). Analysis of single cell fate in response to Taxol and 4-OHT indicated ~67% of cells undergoing apoptosis (Fig. 6E).

Together, these data show that Bcl-XL retrotranslocation is intimately linked to interactions with its BH3-protein partners. Thus, measuring changes in Bcl-XL retrotranslocation is indicative of dynamic variations in apoptotic priming.

## Discussion

Here we define a link between retrotranslocation of anti-apoptotic Bcl-2 proteins and mitochondrial priming. Bcl-XL and Bcl-W retrotranslocate rapidly in unprimed cells, but become stabilised on mitochondria by binding BH3-only proteins. The amount of Bcl-XL in the stable mitochondrial fraction correlated with apoptotic priming. Measuring single cell variation in Bcl-XL retrotranslocation predicted the fractional response to a subsequent pro-apoptotic signal, and provides a non-destructive method for following dynamic changes mitochondrial priming.

Multidomain Bcl-2 proteins constantly shuttle between the cytosol and mitochondria in healthy cells. Bax, Bcl-XL, and Bcl-W all retrotranslocate rapidly, whilst Bcl-2 and Bak, with more hydrophobic tail anchors, are more stable on the OMM [23, 27]. Bax retrotranslocation has been linked to apoptotic priming [23], slowing when survival signals were inhibited, causing it to accumulate on mitochondria and increase priming. Retrotranslocation increased if signalling was restored. Consistent with this, increased mitochondrial Bax correlates with AML patient response to therapy [40]. Similarly, anti-apoptotic Bcl-2 protein retrotranslocation is linked to apoptotic priming and dynamically shifts with BH3-protein activity.

MOMP is a binary event [41], which protects cells from sublethal levels of caspase activation [42]. However, there is considerable variation within a population in the proportion and timing of individual cells dying, which has implications for fractional killing of cancer cells by chemotherapeutics. However, it is inherently difficult to predict a cells response due to the complexity of the signalling networks involved [43]. BH3-profiling allows phenotypic quantification of priming in cultured cell lines or preparations of primary cancer cells from patients [1, 9, 10]. Using this approach, cells are permeabilized to allow the introduction of BH3-peptides from proapoptotic Bcl-2 proteins and priming quantified using mitochondrial membrane potential sensitive dyes. BH3-profiling allows rapid evaluation of a cell population’s MOMP competency, especially useful when applied to primary cells as it minimises modifying their priming by ex vivo culture. Furthermore, varying the BH3-peptides used provides a snapshot of a population’s dependence on individual anti-apoptotic proteins. However, BH3-profiling is inherently destructive, precluding studying shifts in priming as cells respond to dynamic changes signalling.

In contrast, measuring subcellular protein dynamics is non-destructive. As it is monitored in live cells, changes in priming can be identified that by themselves do not reach the threshold for MOMP, but are biologically significant for how cells respond to further stress. As GFP-Bcl-XL is promiscuous for BH3-protein binding, its retrotranslocation is a surrogate measure of priming. This was validated with conditionally active BadER^Tam^, showing dynamic changes in priming whilst cells remained viable. Competency for MOMP was confirmed with etoposide or Taxol. Thus, the retrotranslocation of GFP-Bcl-XL provides a non-destructive assay to model dynamic changes in priming. GFP-Bcl-XL retrotranslocation also showed marked heterogeneity between single cells in a population, and this variation predicted the proportion of cells that would die would following a subsequent chemotherapeutic challenge. Thus, GFP-Bcl-XL retrotranslocation can provide a sensitive and non-destructive way to experimentally interrogate dynamic priming in live cells as they respond to changes in their microenvironment.

A question that remains is what role retrotranslocation of Bcl-2 proteins plays in their function. One model is that Bcl-XL removes Bax and, to a lesser extent, Bak by co-retrotranslocation [22, 27]. In contrast, other data suggests that the Bcl-XL/Bax complexes stabilise both proteins on mitochondria [23, 31]. Bcl-XL can disrupt Bax oligomers, promoting their disassembly into dimers that are still within the membrane [44]. Our results suggest that binding of Bcl-XL to either BH3-only or multi-domain pro-apoptotic proteins results in more stable complexes at mitochondria, albeit to varying degrees. One might hypothesise that stabilisation of complexes containing pro- and anti-apoptotic proteins on mitochondria acts to recruit the pro-apoptotic Bcl-2 members whilst at the same time inhibiting them. Manipulating the dynamics of these proteins and examining them using live imaging should provide important insights into the complexities of apoptotic regulation.

## Methods

### Antibodies, drugs, and immunoblotting

The following antibodies were used: anti-cleaved caspase 3 (R&D Systems); anti-GFP, (Invitrogen); anti-oestrogen receptor alpha (Santa Cruz); anti-V5 (Serotec); anti-mtHsp70 (Affinity Bioreagents); anti-Vinculin (Sigma), anti-pBad phospho-S112 (New England Biolabs #2921S), anti-Bad (Cell Signalling Technology #9239T) secondary antibodies (Jackson Labs). Etoposide, paclitaxel, and 4-hydroxy tamoxifen were obtained from Sigma.

For immunoblotting, proteins were separated by SDS PAGE. Following transfer to nitrocellulose, membranes were incubated with primary antibodies. Proteins were detected with IrDye 800 and 680 conjugated secondary antibodies (Rockland cat# 610-745-124 and LiCor, cat# 926-680-23D), which were detected using an Odyssey CLx imager (LiCor). The full-length blots are shown in the Supplementary Data.

### Expression constructs

For the ∆TM variants, Bcl-2, Bcl-W, Bcl-XL, and Mcl-1 were truncated by introducing a stop codon upstream of the hydrophobic transmembrane domain *via* site-directed mutagenesis (T219, T172, W213, N330 respectively). Bcl-XLR139D was generated *via* site-directed mutagenesis on GFP-BclXL. Mutagenesis was performed using either QuikChange Lightning Site-Directed Mutagenesis Kit (Agilent) or by Gibson assembly (New England Biolabs) within the full-length versions in pEGFP or pCDH vectors. mCherry-tagged tBid, BimEL and Bad, along with the BH3-domain variants, were generous gifts from David Andrews (McMaster University). mCherry-Puma and Noxa were generated by PCR amplification of the coding sequences and cloning into mCherryC1.

Lentivirus expression vectors were generated using pCDH-EF1-MCS-T2A (SystemBiosciences). GFP and paGFP-Bcl2 proteins were inserted downstream of the T2A sequence. For the paGFP variants, H2B-mRFP was inserted upstream of the T2A sequence [23]. BadER^Tam^ was generated by cloning the coding sequence for Bad into pCDH-EF1-T2A-GFP-BclXL, and subsequently inserting the coding sequence for the oestrogen receptor hormone-binding domain [45] in frame between Bad and the T2A sequence. Site-directed mutagenesis was used to substitute the codons for S112 and S136 to those for alanine.

### Cell culture

MCF10A and MDA-MB-231 cells were obtained from ATCC. Bax/Bak double knock out MEFs were originally obtained from Stanley Korsmeyer (Dana Farber). MEFs and MDA-MB-231 were grown in DMEM (Lonza) supplemented with 10% FBS (Labtech) and 1% penicillin/streptomycin (Sigma). MCF10A cells were grown in DMEM-F12 (Lonza) supplemented with 5% Horse Serum (Biosera), 25 ng/ml EGF, 0.5mg/ml hydrocortisone, 100ng/ml Cholera Toxin, 10 µg/ml insulin, 1% penicillin/streptomycin (Sigma). All lines were maintained at 37 °C with 5% CO_2_. All cells were maintained in 10 cm dishes (Corning) and passaged when appropriate.

For transient transfections, cells were seeded into 35 mm glass bottom dishes (MatTek) 24 h pre-transfection to ensure approximately 70% confluency the next day. Cells were transfected using TransIT-X2 reagent (Mirus) as per manufacturer’s instructions.

For stable viral infection, cells were seeded into a six-well culture plate (Corning) 24 h prior to infection to ensure approximately 40% confluency for infection. Cells were infected in full growth medium containing 2 µg/ml Polybrene (Millipore) and the appropriate concentration of virus. The cells were then incubated overnight at 37 °C before adding fresh growth medium. Cells were passaged for two weeks and stably expressing cells selected by fluorescence-activated cell sorting (FACS).

### Live cell imaging

Prior to imaging, cells were washed twice in PBS and 2 ml of live-imaging medium (Ham’s-F12 supplemented with 10% FBS (Lonza), 1% penicillin/streptomycin, and 25mM HEPES (Sigma)) was added to each dish before incubating in the microscope chamber at 37 °C for a minimum of 1 h prior to imaging. 4-OHT (Sigma) was added to cells at a concentration of 10 nM for 1 h before imaging. Images were captured using a 3i Zeiss confocal system with a CSU-X1 Spinning Disc (Yokogawa), using a 63×/1.40 Plan Apochromat objective (Zeiss), an Evolve EM-CDD camera (Photometrics), and a motorised XYZ stage (Intelligent Imaging Innovations) driven by Marianas hardware. Images were captured using Slidebook 5.0 software (Intelligent Imaging Innovations).

For FRAP, a region of interest (ROI) was selected within the cytosol of a cell and photobleached (10 ms, 100% laser power, 488 nm for GFP, 594 nm for mCherry). Images were taken at 5 s intervals for 30 s pre-bleach and then post-bleaching. For GFP photoactivation, a ROI was selected within the cytosol and photoactivated for 10 ms using a 405 nm laser at 100% power. Images were analysed using Image J software. Briefly, background was subtracted using a rolling ball radius of 50 before measuring levels of fluorescence within the bleached ROI in each sequential image. Data were normalised to 100% fluorescence pre-bleach (FRAP) or 100% fluorescence post-photoactivation and a one-phase association or dissociation curve fitted.

### Apoptosis assays and immunofluorescent imaging

Cells were immunostained for active caspase 3 as previously described [23]. For quantitation of apoptosis in cells co-expressing mCherry BH3-proteins and the ∆TM mutants, cells were immunostained for mCherry and GFP, and nuclear morphology assessed following staining with DAPI. Images were taken with a 63× (NA 1.4) Plan Apochromat objective on a Zeiss Axio Imager M2 using ImageJ.

For analysis of endogenous Bad, MCF10A cells were cultured either in complete growth media or serum-free growth media for 24 h. After 24 h, media was replaced for 1h, either with complete growth media (serum and recovery conditions) or serum-free growth media (serum starvation condition). After 1 h, cells were fixed in 4% PFA and stained for DAPI alongside either anti-pBad S112 or total Bad. Cells were imaged using an EVOS M7000 using a 40× Plan Fluorite 0.75NA objective (for quantitative analysis) or a Zeiss Axioplan 2 using a 63× 1.4NA Plan Apochromat objective (for images). Fiji was used to determine the corrected total cell fluorescence of a minimum of 100 individual cells per condition.

### Single cell fate profiling

Cells were cultured in 24-well tissue culture plates, and treated with 1 μM Taxol, 5 μM ABT-737, or 10 μM 4-OHT as detailed in text, and imaged at 15 min intervals for 50 h, maintained at 37 °C and 5% CO_2_. Images were acquired on an AS MDW live cell imaging system (Leica) in brightfield using a 20× HC Plan Fluotar objective, using point visiting to allow multiple positions to be captured within the same time course. Image stacks were analysed using ImageJ, single cells identified and followed manually to determine fate.

### Statistical analysis

Details of statistical analysis used are provided in each figure legend. All analysis was undertaken using GraphPad Prism.

## Supplementary information

Supplementary data

Marked up manuscript file

SupplementaryMovie 1

SupplementaryMovie 2

SupplementaryMovie 3

SupplementaryMovie 4

SupplementaryMovie 5

SupplementaryMovie 6

SupplementaryMovie 7

SupplementaryMovie 8

Change of authorship request form

author checklist

author checklist

## Data Availability

All data generated or analysed during this study are included in this published article and its supplementary information files.
