# Supplementary material for: Apoptotic priming is defined by the dynamic exchange of Bcl-2 proteins between mitochondria and cytosol
Source: Cell Death Differ. 2022 May 18;29(11):2262–74. doi: 10.1038/s41418-022-01013-z (PMC9613888; doi:10.1038/s41418-022-01013-z)

Apoptotic priming is defined by the dynamic exchange of Bcl-2 proteins between mitochondria and cytosol.

Louise E. King, Ricardo Rodriguez-Enriquez, Robert Pedley, Charlotte E.L. Mellor, Pengbo Wang, Egor Zindy, Michael R.H. White, Keith Brennan, and Andrew P. Gilmore.

Supplementary data

Supplementary figure legends.

Figure S1. Mitochondrial stabilisation of anti-apoptotic Bcl-2 proteins is required for their function.

DKO MEF transiently expressing GFP-or paGFP-tagged Bcl-XL and Bcl-2 were subjected to FRAP or photoactivation analysis.

A. Example image sequences from FRAP analysis of GFP-Bcl-XL, GFP-Bcl-2 and GFP-BclW. Area of photobleaching is indicated by the yellow box in the pre-bleach frame. Scale bar - 10μm.

B. Example image sequences from photactivation analysis from cells expressing paGFP-Bcl-XL and paGFP-Bcl-2. Cells were co-expressing mRFP-H2B to allow identification of transfected cells. Area of photoactivation is indicated by the yellow box. Scale bar - 10μm.

C. GFP-tagged Bcl-2, Bcl-XL and Bcl-W were modified by inserting stop codons immediately upstream of the transmembrane (TM) tail anchor sequences (highlighted in blue), indicated by the red Z in the protein sequence shown.

Figure S2. Bcl-XL is stabilised on mitochondria by BH3-domain specific interactions.

A. FRAP analysis of GFP-Bcl-XL in DKO MEFs transiently expressing the indicated mCherry-tagged wildtype tBid or the indicated BH3-doamin variants. GFP-Bcl-XL was photobleached in the yellow ROI and imaged every 5 seconds. Fluorescent intensity was analysed within the ROI and normalised to 100% pre-bleaching. The data were fitted to a one-phase exponential association +/- SEM.

B. FRAP analysis of GFP-tagged Bcl-XL as in A. DKO MEFs transiently expressing the indicated mCherry-tagged BimEL variants were photobleached in the yellow ROI and imaged every 5 seconds.

C. Wild-type MEFs were transiently transfected with either mCherry or the indicated mCh tBid and Bim variants and cultured for 18 hrs. Cells were then fixed in paraformaldehyde and apoptosis was quantified by immunostaining for activated caspase 3. Error bars represent standard deviation and data was analysed by one-way ANOVA and Šídák's post hoc test. ** = $p<0.005$, *** = $p<0.0005$.

Figure S3. Serum starvation reduces GFP-BclXL FRAP recovery.

A. Example image sequence from Fig. 3B. MCF10A mammary epithelial cells stably expressing GFP-Bcl-XL were either grown in complete media or serum starved. GFP-Bcl-XL was photobleached in the yellow ROI and imaged every 5 seconds. Scale bars represent 10μm.

B. MCF10A cells cultured either in complete growth media or serum-free growth media for 24h. After 24h, media was replaced for 1h with either media with serum and GFs or fresh serum-free growth media. Cells were fixed and immunostained for phosphorylated Bad serine 112 and DAPI. Scale bars represent 10μm.

C. MCF10A cells treated as in B. were immunostained for mitochondrial Hsp70 (mtHsp70) and total Bad, along with DAPI. Scale bars represent 10μm.

Figure S4. Bcl-XL retrotranslocation indicates dynamic changes in BH3-only protein activity within live cells.

A. Representative image sequences from Fig.4C. MCF10A cells stably expressing BadER^{Tam}/GFP-BclXL, and treated with ethanol or 4-OHT, or transiently expressing mCh-Bad. GFP-Bcl-XL was photobleached in the yellow ROI. Scale bar represents 10μm.

B. 4-OHT does not alter GFP-Bcl-XL retrotranslocation in the absence of BadER^{Tam}. FRAP carried out on MCF10As expressing either GFP-Bcl-XL alone or BadER^{Tam}/GFP-BclXL treated with either ethanol (-4-OHT) or 4-OHT. Non-linear regression was carried out and fluorescence recovery plotted. Only BadER^{Tam}/GFP-BclXL cells were affected by 4-OHT treatment. Values represent data from 2 independent experiments. Error bars represent SD and data was analysed via one-way ANOVA. **** = p<0.001.

C. BadERTam-GFP-Bcl-XL MCF10A cells were subjected to FRAP (day 0) before being treated with either ethanol (EtOH) or 4-OHT for 24 hours. Treatment was washed out and a second round of FRAP was carried out immediately afterwards (day 1). Subsequent FRAP was performed 3 days and 6 days post-washout. The average % fluorescence recovery was calculated for each condition. Values represent 60 cells per condition. Error bars represent SD.

Figure S5. GFP-Bcl-XL retrotranslocation dynamics are distinct between MODE1 and MODE2 inhibition.

A. Bax/Bak DKO MEFs stably infected with the lentivirus expressing BadER^{Tam} and GFP-Bcl-XL were transiently transfected with expression vectors for mCh-tBid or mCh-tBdG94E. Cells were either untreated or treated with 4-OHT. FRAP analysis was performed on the GFP-Bcl-XL and the percentage recovery calculated.

B. FRAP was performed on the mCh-tBid or mCh-tBidG94E simultaneously on the same cells analysed in A. The percentage recovery was calculated.

C. Bax/Bak DKO MEFs stably expressing BadER^{Tam} and GFP-Bcl-XL, either unstransfected or transiently transfected with an expression vector for mCh-Bak, were untreated or treated with 4-OHT as indicated. FRAP analysis was performed on the GFP-Bcl-XL and the percentage recovery calculated.

D. The same cells in C. transfected with mCh-Bak were simultaneously analysed for mCherry FRAP and percentage recovery calculated.

Fig S6. Single cell variations in GFP-Bcl-XL dynamics are cell autonomous and correlate with Bad phosphorylation.

A. MCF10A cells were serum starved and treated as in Fig.S3B. Cells were fixed and immunostained with anti-phosphorylated Bad serine 112. Cells were imaged using an EVOS M7000 microscope and the intensity of immunostaining quantified using Image J. The histogram on the left shows the number of cells in each condition that fall within each binned level of intensity. The plot on the right shows the single cell distributions of phosphorylated-Bad staining under each condition. Data were na.aysed using one-way analysis of variance. **** = $p<0.001$.

B. Immunofluorescence of the mixed parental line and ten single cell clones of BadER^{Tam}/GFP-BclXL expressing MCF10A cells. All show the similar mitochondrial distribution of GFP-Bcl-XL.

C. FRAP analysis from Fig.3B, fitted to a one-phase exponential association +/- SEM.

Figure S7. The apoptotic response of parental MDA-MB-231 cells is not affected by 4-OHT.

Parental wild-type MDA-MB-231 cells were treated with DMSO or Taxol, in the presence or absence of 4-OHT, and cell fate followed as in Fig6B. The addition of 4-OHT has no significant effect of the fate of the parental cell line.

Supplementary movies.

Supplementary Movie 1. FigS1B paGFP-Bcl-Xl

Supplementary Movie 2. FigS1B paGFP-Bcl-2

Supplementary Movie 3. FigS1B paGFP-Bcl-W

Supplementary Movie 4. Fig2A paGFP-Bcl-Xl plus mCh-tBid

Supplementary Movie 5. Fig2A paGFP-Bc-lXl plus mCh-Bim

Supplementary Movie 6. Fig2A paGFP-Bcl-Xl plus mCh-Puma

Supplementary Movie 7. Fig2A paGFP-Bcl-Xl plus mCh-Bad

Supplementary Movie 8. Fig2A paGFP-Bcl-Xl plus mCh-Noxa

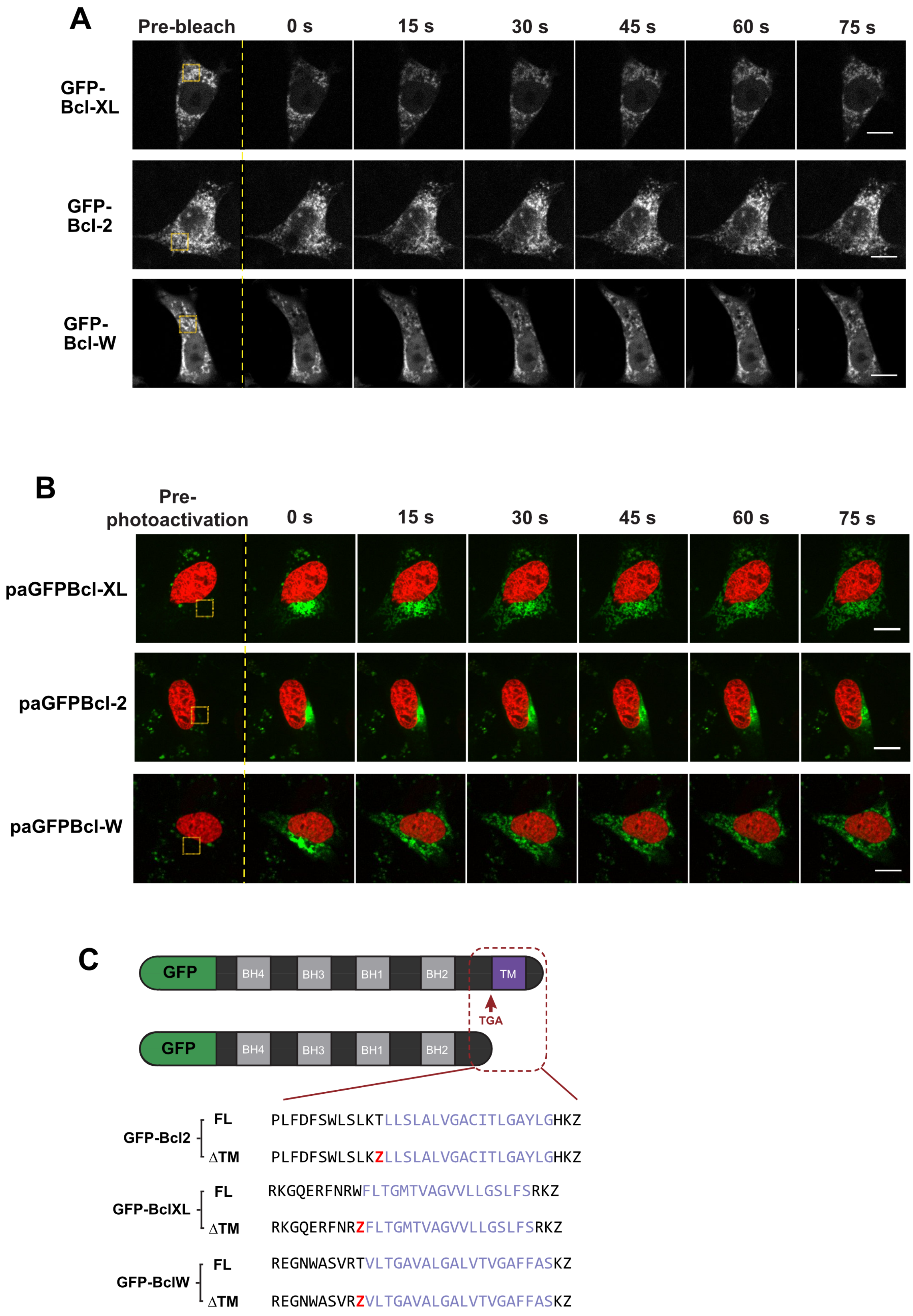
A
Pre-bleach
0 s
15 s
30 s
45 s
60 s
75 s
GFP-Bcl-XL
GFP-Bcl-2
GFP-Bcl-W
B
Pre-photoactivation
0 s
15 s
30 s
45 s
60 s
75 s
paGFPBcl-XL
paGFPBcl-2
paGFPBcl-W
C
GFP
BH4
BH3
BH1
BH2
TM
TGA
GFP
BH4
BH3
BH1
BH2
GFP-Bcl2
FL PLFDFSWLSLKTLLSLALVGACITLGAYLGHKZ
ΔTM PLFDFSWLSLKZLLSLALVGACITLGAYLGHKZ
GFP-BclXL
FL RKGQERFNRWFLTGMTVAGVVLLGSLFSRKZ
ΔTM RKGQERFNRZFLTGMTVAGVVLLGSLFSRKZ
GFP-BclW
FL REGNWASVRTVLTGAVALGALVTVGAFFASKZ
ΔTM REGNWASVRZVLTGAVALGALVTVGAFFASKZ

Figure S1

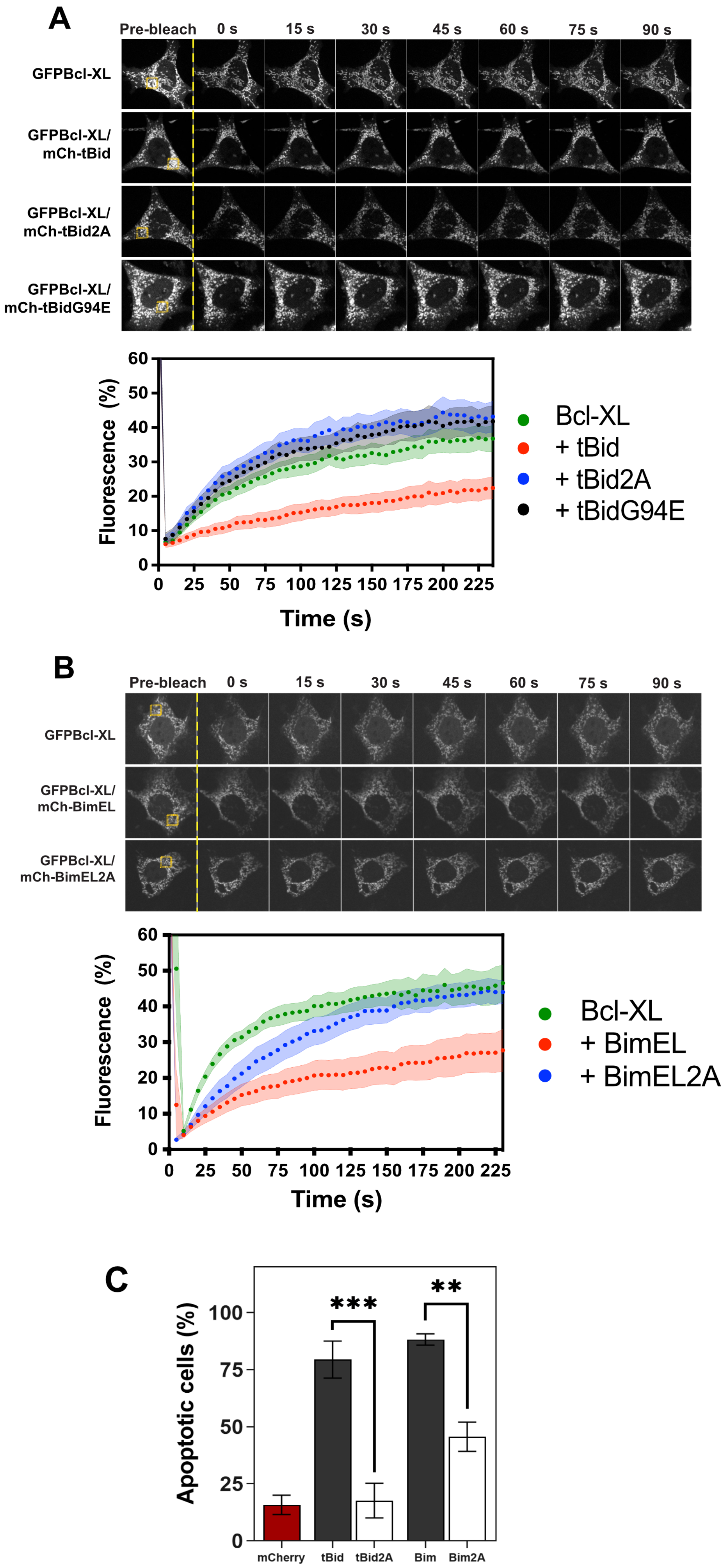
A
Pre-bleach
0 s
15 s
30 s
45 s
60 s
75 s
90 s
GFPBcl-XL
GFPBcl-XL/ mCh-tBid
GFPBcl-XL/ mCh-tBid2A
GFPBcl-XL/ mCh-tBidG94E
Fluorescence (%)
Time (s)
Bcl-XL
+ tBid
+ tBid2A
+ tBidG94E
B
Pre-bleach
0 s
15 s
30 s
45 s
60 s
75 s
90 s
GFPBcl-XL
GFPBcl-XL/ mCh-BimEL
GFPBcl-XL/ mCh-BimEL2A
Fluorescence (%)
Time (s)
Bcl-XL
+ BimEL
+ BimEL2A
C
Apoptotic cells (%)
***
**
mCherry
tBid
tBid2A
Bim
Bim2A

Figure S2

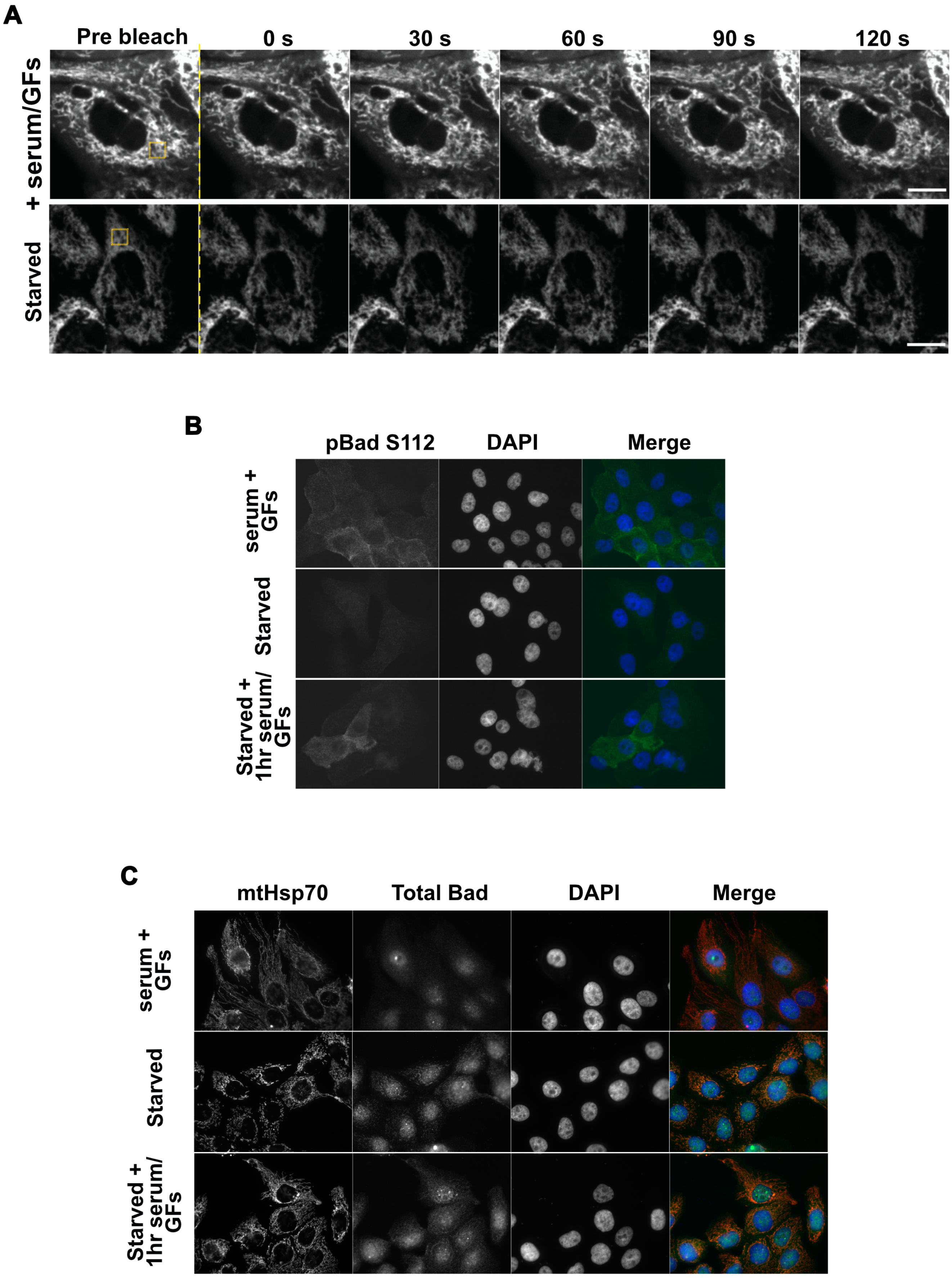
A
Pre bleach
0 s
30 s
60 s
90 s
120 s
+ serum/GFs
Starved
B
pBad S112
DAPI
Merge
serum + GFs
Starved
Starved + 1hr serum/ GFs
C
mtHsp70
Total Bad
DAPI
Merge
serum + GFs
Starved
Starved + 1hr serum/ GFs

Figure S3

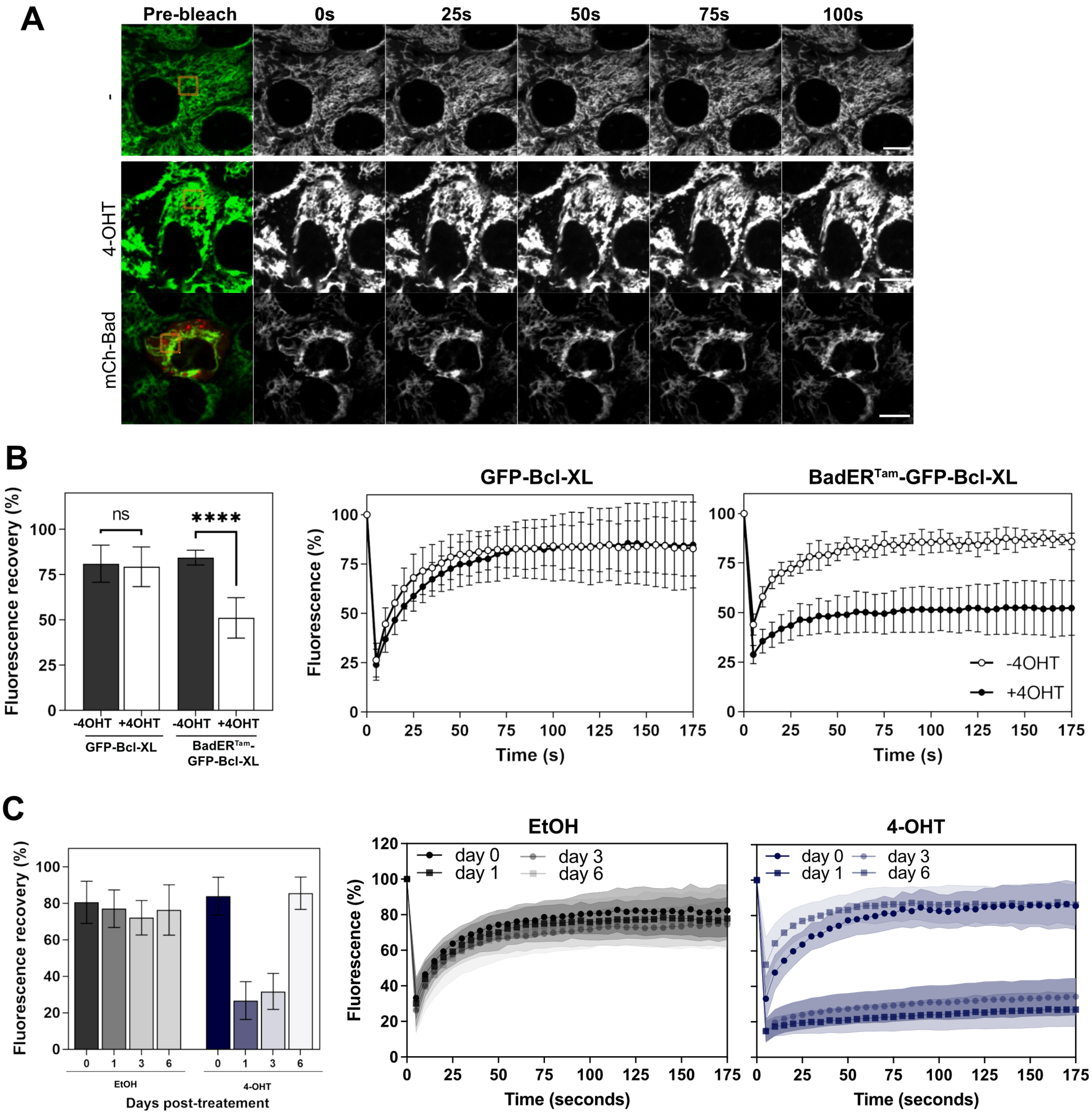
A
Pre-bleach
0s
25s
50s
75s
100s
-
4-OHT
mCh-Bad
B
GFP-Bcl-XL
BadER^Tam-GFP-Bcl-XL
Fluorescence recovery (%)
ns
****
-4OHT
+4OHT
GFP-Bcl-XL
BadER^Tam-GFP-Bcl-XL
Fluorescence (%)
Time (s)
C
EtOH
4-OHT
day 0
day 1
day 3
day 6
Fluorescence recovery (%)
Days post-treatement
Fluorescence (%)
Time (seconds)

Figure S4

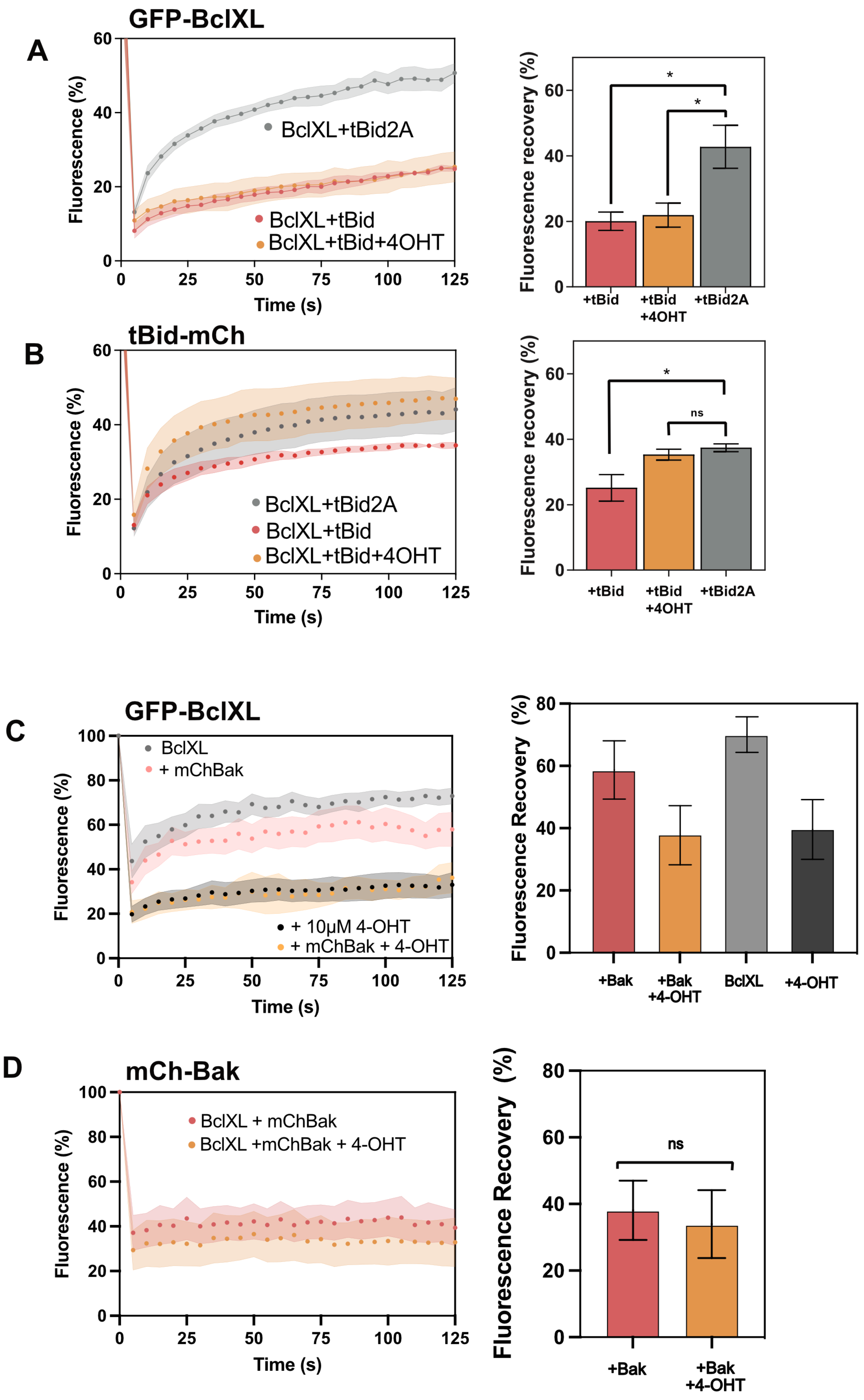

A
GFP-BclXL
Fluorescence (%)
Time (s)
BclXL+tBid2A
BclXL+tBid
BclXL+tBid+4OHT
Fluorescence recovery (%)
*
*
+tBid
+tBid +4OHT
+tBid2A
B
tBid-mCh
BclXL+tBid2A
BclXL+tBid
BclXL+tBid+4OHT
ns
C
GFP-BclXL
BclXL
+ mChBak
+ 10µM 4-OHT
+ mChBak + 4-OHT
Fluorescence Recovery (%)
+Bak
+Bak +4-OHT
BclXL
+4-OHT
D
mCh-Bak
BclXL + mChBak
BclXL +mChBak + 4-OHT

Figure S5

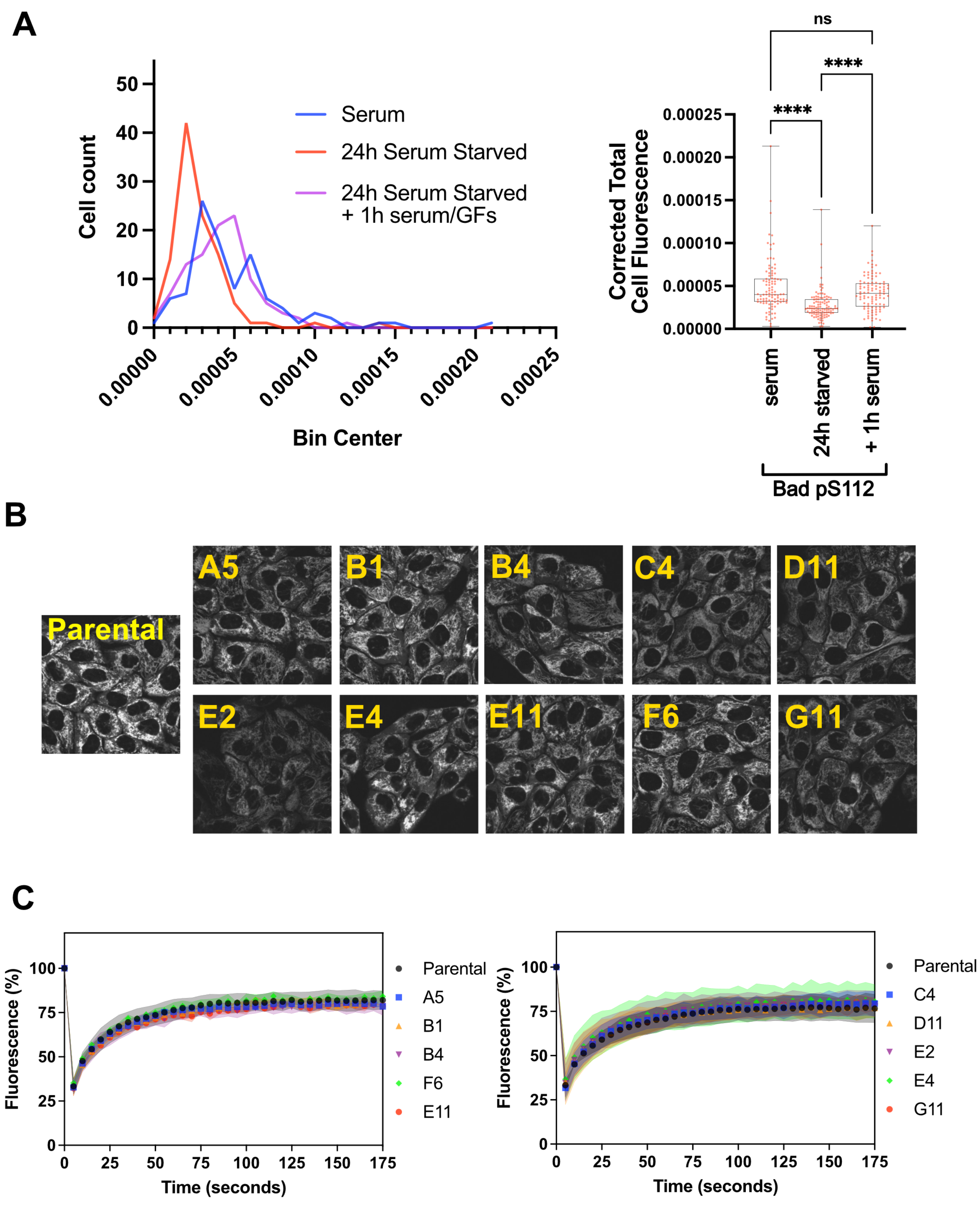
A
Cell count
Serum
24h Serum Starved
24h Serum Starved
+ 1h serum/GFs
Bin Center
Corrected Total
Cell Fluorescence
ns
****
****
serum
24h starved
+ 1h serum
Bad pS112
B
Parental
A5
B1
B4
C4
D11
E2
E4
E11
F6
G11
C
Fluorescence (%)
Time (seconds)
Parental
A5
B1
B4
F6
E11
Parental
C4
D11
E2
E4
G11

Figure S6

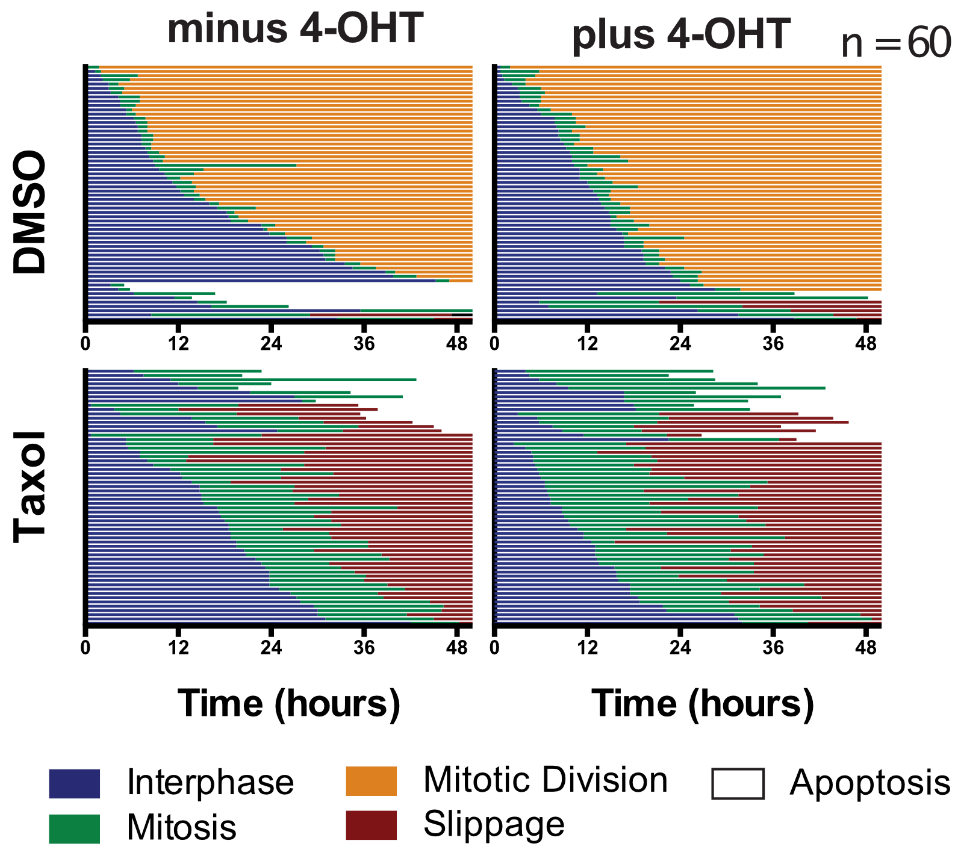

minus 4-OHT
plus 4-OHT
n = 60
DMSO
Taxol
0
12
24
36
48
Time (hours)
Time (hours)
Interphase
Mitosis
Mitotic Division
Slippage
Apoptosis

Figure S7

Supplementary data: Full length western blots from main paper figures
Cropped area shown in main figure is indicated in yellow

Full length western blot relating to Figure 1C

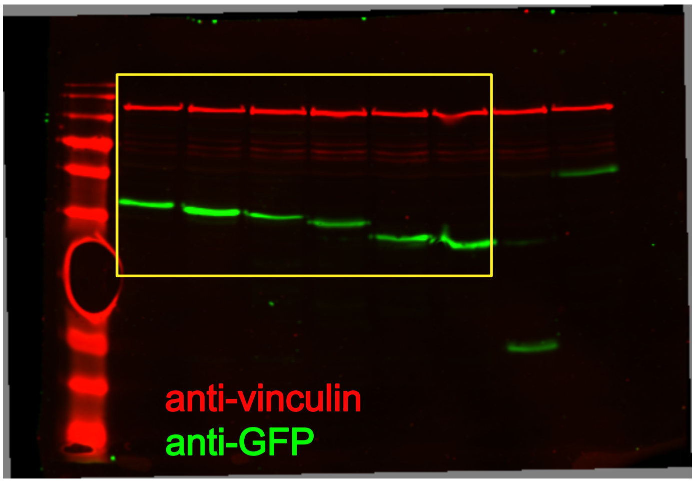

Full length western blot relating to Figure 5A

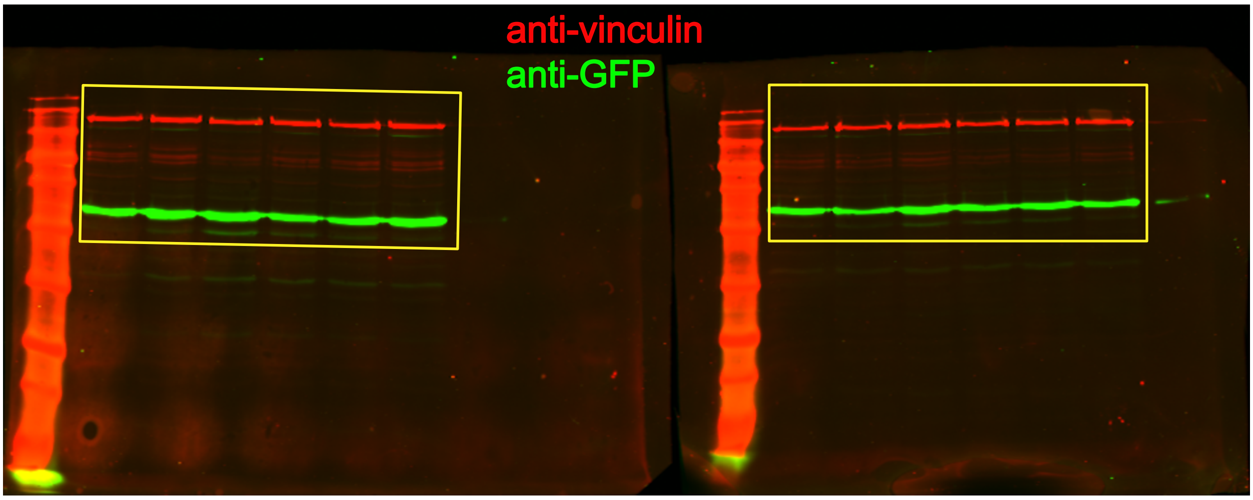

Supplement: Supplementary file 1 — Supplementary data [file 41418_2022_1013_MOESM1_ESM.pdf]
