# Supplementary material for: Apoptotic priming is defined by the dynamic exchange of Bcl-2 proteins between mitochondria and cytosol
Source: Cell Death Differ. 2022 May 18;29(11):2262–74. doi: 10.1038/s41418-022-01013-z (PMC9613888; doi:10.1038/s41418-022-01013-z)
Supplement: Supplementary file 11 — Change of authorship request form [file 41418_2022_1013_MOESM11_ESM.pdf]

SPRINGER NATURE

Change of authorship request form - Journals (pre-acceptance)

Important information. Please read.

- This form should be used by authors to request any change in authorship (adding/deleting authors) including changes in corresponding authors. This form should not be used for name changes. Please fully complete all sections. Use black ink and block capitals and provide each author's full name with the given name first followed by the family name.

- By signing this declaration, all authors guarantee that the order of the authors are in accordance with their scientific contribution, if applicable as different conventions apply per discipline, and that only authors have been added who made a meaningful contribution to the work.

- Please note, in author collaborations where there is formal agreement for representing the collaboration, it is sufficient for the representative or legal guarantor (usually the corresponding author) to complete and sign the Authorship Change Form on behalf of all authors, **next to the added/removed author(s)**. **(Complete Section 3, followed by Section 6.)**
  In author collaborations where there is no formal agreement for representing the collaboration and **there are more than 10 authors**, one may sign for all, provided the signer appends correspondence that attests that each of the authors have agreed to the change **and the added/removed authors sign the form**. **(Complete Section 3, followed by Section 6.)**

- Please note, we cannot investigate or mediate any authorship disputes. If you are unable to obtain agreement from all authors (including those who you wish to be removed) you must refer the matter to your institution(s) for investigation. Please inform us if you need to do this.

- If you are not able to return a fully completed form within **30 days** of the date that it was sent to the author requesting the change, we may have to withdraw your manuscript. We cannot publish manuscripts where authorship has not been agreed by all authors (including those who have been removed).

- Incomplete forms will be rejected.

- Please return/upload this form, fully completed, to the Journals Editorial Office. The Journal and/or Publisher will consider the information you have provided to decide whether to approve the proposed change in authorship. We may decide to contact your institution for more information or undertake a further investigation, if appropriate, before making a final decision.

SPRINGER NATURE

Change of authorship request form - Journals (pre-acceptance)

Section 1: Please provide the current title of manuscript

Manuscript ID no.: CDD-21-1679RR

Title: Apoptotic priming is defined by the dynamic exchange of Bcl-2 proteins between mitochondria and cytosol.

Section 2: Please provide the previous authorship, in the order shown on the manuscript before the changes were introduced. Please indicate the corresponding author by adding (CA) behind the name.

	First name(s)	Family name	ORCID or SCOPUS id, if available
1st author	Louise	King	0000-0003-2453-8887
2nd author	Ricardo	Rodriguez-Enriquez	
3rd author	Robert	Pedley	0000-0002-0801-7240
4th author	Pengbo	Wang	
5th author	Egor	Zindy	0000-0003-0033-0002
6th author	Keith	Brennan	0000-0002-9384-125X
7th author	Michael	White	0000-0002-3617-3232
8th author	Andrew	Gilmore (CA)	0000-0002-9988-0436
9th author			
10th author			

Please use an additional sheet if there are more than 10 authors.

Section 3: Please provide a justification for change. Please use this section to explain your reasons for changing the authorship of your manuscript, e.g. what necessitated the change in authorship? Please refer to the (journal) policy pages for more information about authorship. Please explain why omitted authors were not originally included and/or why authors were removed on the submitted manuscript.

SPRINGER NATURE ---

Springer Nature is one of the world's leading global research, educational and professional publishers, created in May through the combination of Nature Publishing Group, Palgrave Macmillan, Macmillan Education and Springer Science+Business Media.

SPRINGER NATURE

Change of authorship request form - Journals (pre-acceptance)

Charlotte Mellor was added to the author list as she undertook some of the revisions requested by the reviewers. This was necessary as some of the original authors were no longer in the lab.

Section 4: Proposed new authorship. Please provide your new authorship list in the order you would like it to appear on the manuscript. Please indicate the corresponding author by adding (CA) behind the name. If the Corresponding Author has changed, please indicate the reason under section 3.

	First name(s)	Family name (this name will appear in full on the final publication and will be searchable in various abstract and indexing databases)	Affiliated institute	E-mail address
1st author	Louise	King	University of Manchester	louiseking090@gmail.com
2nd author	Ricardo	Rodriguez-Enriquez	University of Manchester	Ricardo.Rodriguez-Enriquez@STJUDE.ORG
3rd author	Robert	Pedley	University of Manchester	robert.pedley@manchester.ac.uk
4th author	Charlotte	Mellor	University of Manchester	charlotte.mellor@postgrad.manchester.ac.uk
5th author	Pengbo	Wang	University of Manchester	Pengbo.Wang@cruk.manchester.ac.uk
6th author	Egor	Zindy	University of Manchester	egor.zindy@ulb.ac.be
7th author	Michael	White	University of Manchester	mike.white@manchester.ac.uk
8th author	Keith	Brennan	University of Manchester	Keith.Brennan@manchester.ac.uk
9th author	Andrew	Gilmore (CA)	University of Manchester	agilmore@manchester.ac.uk
10th author				

Please use an additional sheet if there are more than 10 authors.

SPRINGER NATURE ---

Springer Nature is one of the world's leading global research, educational and professional publishers, created in May
through the combination of Nature Publishing Group, Palgrave Macmillan, Macmillan Education and Springer Science+Business Media.

SPRINGER NATURE

Change of authorship request form - Journals (pre-acceptance)

Section 5: Author contribution, Acknowledgement and Disclosures. Please use this section to provide a new disclosure statement and, if appropriate, acknowledge any contributors who have been removed as authors and ensure you state what contribution any new authors made (if applicable per the journal or book (series) policy). Please ensure these are updated in your manuscript - after approval of the change(s) - as our production department will not transfer the information in this form to your manuscript.

New acknowledgements:

We thank Peter March and Roger Meadows for assistance with imaging, Mike Jackson for FACS, David Andrews for providing mCh-BH3 proteins, and members of the Wellcome Trust Centre for Cell Matrix Research for discussion and comments on the manuscript.

New Disclosures (financial and non-financial interests, funding):

This work was supported by grants from Breast Cancer Now and Cancer Research UK. R.R-E was supported by Connacyt. L.E.K and C.E.L.M were supported by MCRC-CRUK training awards. R.P. was supported by a PhD scholarship funded by John and Janet Hartley. The Wellcome Trust Centre for Cell-Matrix Research is supported by core funding from the Wellcome Trust (088785/Z/09/Z). This work was supported by the Wellcome Institutional Strategic Support Fund award (204796/Z/16/Z). The Bioimaging Facility microscopes used in this study were purchased with grants from BBSRC, The Wellcome Trust and the University of Manchester Strategic Fund.

New Author Contributions statement (if applicable per the journal policy):

L.E.K, R.R-E. and C.E.L.M performed imaging experiments and analysed the data. R.P. performed the mitotic arrest and apoptosis assays. P.W. and R.R-E. generated lentiviral expression constructs. E.Z. worked on image analysis. A.P.G conceived the project. K.B., M.R.H.W. and A.P.G. supervised the research, and wrote the manuscript with the help of all the authors.

State 'Not applicable' if there are no new authors.

SPRINGER NATURE ---

Springer Nature is one of the world's leading global research, educational and professional publishers, created in May
through the combination of Nature Publishing Group, Palgrave Macmillan, Macmillan Education and Springer Science+Business Media.

SPRINGER NATURE

Change of authorship request form - Journals (pre-acceptance)

Section 6: Declaration of agreement. *All* authors, unchanged, new and removed *must* sign this declaration.
(NB: Please print the form, (docu)-sign and return/upload a scanned copy. Please note that signatures that have been inserted as an image file are acceptable as long as it is handwritten. Typed names in the signature box are unacceptable.) *** Please delete as appropriate. Delete all of the bold if you were on the original authorship list and are remaining as an author.**

	First name	Family name		Signature	Date
1st author	Louise	King	I agree to the proposed new authorship shown in section 4 **/and the addition/removal*of my name to the authorship list** /and the proposed change in corresponding author		20th April 2022
2nd author	Ricardo	Rodriguez-Enriquez	I agree to the proposed new authorship shown in section 4 **/and the addition/removal*of my name to the authorship list** /and the proposed change in corresponding author		20th April 2022
3rd author	Robert	Pedley	I agree to the proposed new authorship shown in section 4 **/and the addition/removal*of my name to the authorship list** /and the proposed change in corresponding author		19.4.22
4th authors	Charlotte	Mellor	I agree to the proposed new authorship shown in section 4 **/and the addition/removal*of my name to the authorship list** /and the proposed change in corresponding author		20th April 2022
5th author	Pengbo	Wang	I agree to the proposed new authorship shown in section 4 **/and the addition/removal*of my name to the authorship list** /and the proposed change in corresponding author		20.04.2022
6th author	Egor	Zindy	I agree to the proposed new authorship shown in section 4 **/and the addition/removal*of my name to the authorship list** /and the proposed change in corresponding author		19th April 2022

SPRINGER NATURE ---
Springer Nature is one of the world's leading global research, educational and professional publishers, created in May
through the combination of Nature Publishing Group, Palgrave Macmillan, Macmillan Education and Springer Science+Business Media.

7th author	Michael	White	I agree to the proposed new authorship shown in section 4 **/and the addition/removal*of my name to the authorship list** /and the proposed change in corresponding author		21 April 2022
8th author	Keith	Brennan	I agree to the proposed new authorship shown in section 4 **/and the addition/removal*of my name to the authorship list** /and the proposed change in corresponding author		22nd April 2022
9th author	Andrew	Gilmore (CA)	I agree to the proposed new authorship shown in section 4 **/and the addition/removal*of my name to the authorship list** /and the proposed change in corresponding author		19th April 2022

Please use an additional sheet if there are more than 10 authors.

In case of author collaborations with formal agreement:

	Name of consortium/consortia	First name	Family name		Signature	Date
Representative/legal guarantor				I agree to the proposed new authorship shown in section 4 **/and the addition/removal*of my name to the authorship list** /and the proposed change in corresponding author		

Both added/removed authors should complete the information in the first table under Section 6.

---- End of form ----

SPRINGER NATURE ---

Springer Nature is one of the world's leading global research, educational and professional publishers, created in May through the combination of Nature Publishing Group, Palgrave Macmillan, Macmillan Education and Springer Science+Business Media.
