# Supplementary material for: Apoptotic priming is defined by the dynamic exchange of Bcl-2 proteins between mitochondria and cytosol
Source: Cell Death Differ. 2022 May 18;29(11):2262–74. doi: 10.1038/s41418-022-01013-z (PMC9613888; doi:10.1038/s41418-022-01013-z)
Supplement: Supplementary file 13 — author checklist [file 41418_2022_1013_MOESM13_ESM.pdf]

DECLARATION OF CONTRIBUTIONS TO ARTICLE

ADMC

Manuscript Number: CDD-21-1679

Journal Name: *Cell Death & Differentiation* (the 'Journal')

Proposed Title of the Contribution:

Apoptotic priming is defined by the dynamic exchange of Bcl-2 proteins between (the 'Contribution)

Author(s):

Louise E. King, Ricardo Rodriguez-Enriquez, Robert Pedley, Charlotte E. L. Mellor, (the 'Authors')

For all *CDD* articles, each person named as an author in the published version must be able to show he or she has contributed substantially to the article.

Authorship credit should be based on 1) substantial contributions to conception and design, acquisition of data, or analysis and interpretation of data; 2) drafting the article or revising it critically for important intellectual content; and 3) final approval of the version to be published. Authors should meet conditions 1, 2 and 3.

Any person who cannot be shown to have made a substantial contribution to the article cannot be listed as an author in the final version. The name of any person who is deemed to have made a minor contribution can, however, appear in the Acknowledgments section of the article.

Please complete the table below to indicate the contributions of all named authors to the manuscript.

Author Full Name:	Specification of Contribution to the Manuscript:
Louise E. King	Performed imaging experiments and data analysis. Contributed to
Ricardo Rodriguez-Enriquez	Performed imaging experiments and data analysis. Contributed to
Robert Pedley	Performed mitotic arrest & apoptosis assays and analysed the
Charlotte E. L. Mellor	Performed imaging experiments and data analysis. Contributed to
Pengbo Wang	Generated lentiviral expression constructs. Contributed to
Egor Zindy	Performed image analysis. Contributed to intellectual content of
Michael R.H. White	Supervised research and assisted in the interpretation of data.
Keith Brennan	Supervised research and assisted in the interpretation of data.
Andrew P. Gilmore	Conceived the project. Supervised research and assisted in the

Please complete the table below to indicate the contributions of all named authors to the figures.

Figure 1:

LEK - performed FRAP experiments. RP - performed mutagenesis, IF and apoptosis assay
all authors discussed results and contributed to interpretation

Figure 2:

LEK - performed FLAP experiments.
all authors discussed results and contributed to interpretation

Figure 3:

LEK - performed FRAP experiments for panels A&B. CELM - performed FRAP in panel C. APG - generated Bcl-XLR139D via mutagenesis.
all authors discussed results and contributed to interpretation

Figure 4:

RRE - Generated inducable BadER construct. LEK - performed FRAP experiments.
all authors discussed results and contributed to interpretation

Figure 5:

LEK - performed western blot, and FRAP experiments.
all authors discussed results and contributed to interpretation

Figure 6:

LEK - performed FRAP experiments. RP - performed single cell fate tracking (apoptosis assay)
all authors discussed results and contributed to interpretation

Signed for and on behalf of the Author(s):	Print Name:	Date:
